# Artificial intelligence-assisted clinical decision support for childhood asthma management: A randomized clinical trial

**DOI:** 10.1371/journal.pone.0255261

**Published:** 2021-08-02

**Authors:** Hee Yun Seol, Pragya Shrestha, Joy Fladager Muth, Chung-Il Wi, Sunghwan Sohn, Euijung Ryu, Miguel Park, Kathy Ihrke, Sungrim Moon, Katherine King, Philip Wheeler, Bijan Borah, James Moriarty, Jordan Rosedahl, Hongfang Liu, Deborah B. McWilliams, Young J. Juhn

**Affiliations:** 1 Precision Population Science Lab, Mayo Clinic, Rochester, Minnesota, United States of America; 2 Department of Internal Medicine, Pusan National University Yangsan Hospital, Yangsan, Korea; 3 Department of Pediatric and Adolescent Medicine, Mayo Clinic, Rochester, Minnesota, United States of America; 4 Department of Artificial Intelligence and Informatics, Mayo Clinic, Rochester, Minnesota, United States of America; 5 Division of Biomedical Statistics and Informatics, Mayo Clinic, Rochester, Minnesota, United States of America; 6 Division of Allergic Diseases, Mayo Clinic, Rochester, Minnesota, United States of America; 7 Department of Health Service Research, Mayo Clinic, Rochester, Minnesota, United States of America; 8 Kern Center for the Science of Health Care Delivery, Mayo Clinic, Rochester, Minnesota, United States of America; Universite de Bretagne Occidentale, FRANCE

## Abstract

**Rationale:**

Clinical decision support (CDS) tools leveraging electronic health records (EHRs) have been an approach for addressing challenges in asthma care but remain under-studied through clinical trials.

**Objectives:**

To assess the effectiveness and efficiency of Asthma-Guidance and Prediction System (A-GPS), an Artificial Intelligence (AI)-assisted CDS tool, in optimizing asthma management through a randomized clinical trial (RCT).

**Methods:**

This was a single-center pragmatic RCT with a stratified randomization design conducted for one year in the primary care pediatric practice of the Mayo Clinic, MN. Children (<18 years) diagnosed with asthma receiving care at the study site were enrolled along with their 42 primary care providers. Study subjects were stratified into three strata (based on asthma severity, asthma care status, and asthma diagnosis) and were blinded to the assigned groups.

**Measurements:**

Intervention was a quarterly A-GPS report to clinicians including relevant clinical information for asthma management from EHRs and machine learning-based prediction for risk of asthma exacerbation (AE). Primary endpoint was the occurrence of AE within 1 year and secondary outcomes included time required for clinicians to review EHRs for asthma management.

**Main results:**

Out of 555 participants invited to the study, 184 consented for the study and were randomized (90 in intervention and 94 in control group). Median age of 184 participants was 8.5 years. While the proportion of children with AE in both groups decreased from the baseline (P = 0.042), there was no difference in AE frequency between the two groups (12% for the intervention group vs. 15% for the control group, Odds Ratio: 0.82; 95%CI 0.374–1.96; P = 0.626) during the study period. For the secondary end points, A-GPS intervention, however, significantly reduced time for reviewing EHRs for asthma management of each participant (median: 3.5 min, IQR: 2–5), compared to usual care without A-GPS (median: 11.3 min, IQR: 6.3–15); p<0.001). Mean health care costs with 95%CI of children during the trial (compared to before the trial) in the intervention group were lower than those in the control group (-$1,036 [-$2177, $44] for the intervention group vs. +$80 [-$841, $1000] for the control group), though there was no significant difference (p = 0.12). Among those who experienced the first AE during the study period (n = 25), those in the intervention group had timelier follow up by the clinical care team compared to those in the control group but no significant difference was found (HR = 1.93; 95% CI: 0.82–1.45, P = 0.10). There was no difference in the proportion of duration when patients had well-controlled asthma during the study period between the intervention and the control groups.

**Conclusions:**

While A-GPS-based intervention showed similar reduction in AE events to usual care, it might reduce clinicians’ burden for EHRs review resulting in efficient asthma management. A larger RCT is needed for further studying the findings.

**Trial registration:**

ClinicalTrials.gov Identifier: NCT02865967.

## Introduction

Growing deployments of Electronic Health Records (EHRs) systems have established large practice-based longitudinal patient records causing an increase in the volume of unstructured data (80%) in the currently available health care records [1]. Inefficient and ineffective use of EHRs due to overwhelming volume has led to physician burnout (70% of clinicians reported health information technology [HIT]-related stress) due to increased workload during their limited (e.g.,15–20 min) clinic visit [2–5]. The application of Artificial Intelligence (AI) to health care may potentially address these challenges through AI-assisted clinical decision support (CDS) tools. The National Academy of Medicine suggested delivering high-value care in the personal and social context through science and technology as one of the key directions for the US health care system [6].

The major challenges for better asthma care during the EHRs era is the lack of efficient and effective CDS meaningfully supporting clinicians and their care teams leading to high-value asthma care improving care quality and outcomes while reducing the costs [7–9]. Not surprisingly, some CDS even increased the costs and time for asthma care at the clinic due to providing more services (eg, almost twice as many lasted >1 hour after implementation of CDS) [8]. At present, no augmented AI-assisted CDS tools for streamlining childhood asthma management are available which fully leverage technologies harnessing EHRs. While many AI algorithms have been developed [10–12] and even approved for Software as Medical Device (SaMD) by the FDA [12, 13], few AI algorithms have been tested and shown to have an actual improvement in health outcomes in a randomized clinical trial (RCT) [14, 15].

We developed the Asthma-Guidance and Prediction System (A-GPS), an AI-assisted CDS tool providing 1) a high-level summary of relevant clinical information (eg, asthma care quality, risk factors and outcomes) for each asthmatic patient, 2) machine-learning-based predictive analytics for future asthma exacerbation (AE) and 3) asthma management options to help clinicians make efficient and effective clinical decision-making for optimal asthma management. Here we report results of *an exploratory pragmatic RCT* that assessed the effectiveness and efficiency of intervention via A-GPS on pertinent asthma outcomes in a real-world primary care setting.

## Methods

### Study design and subjects

**Fig 1:**
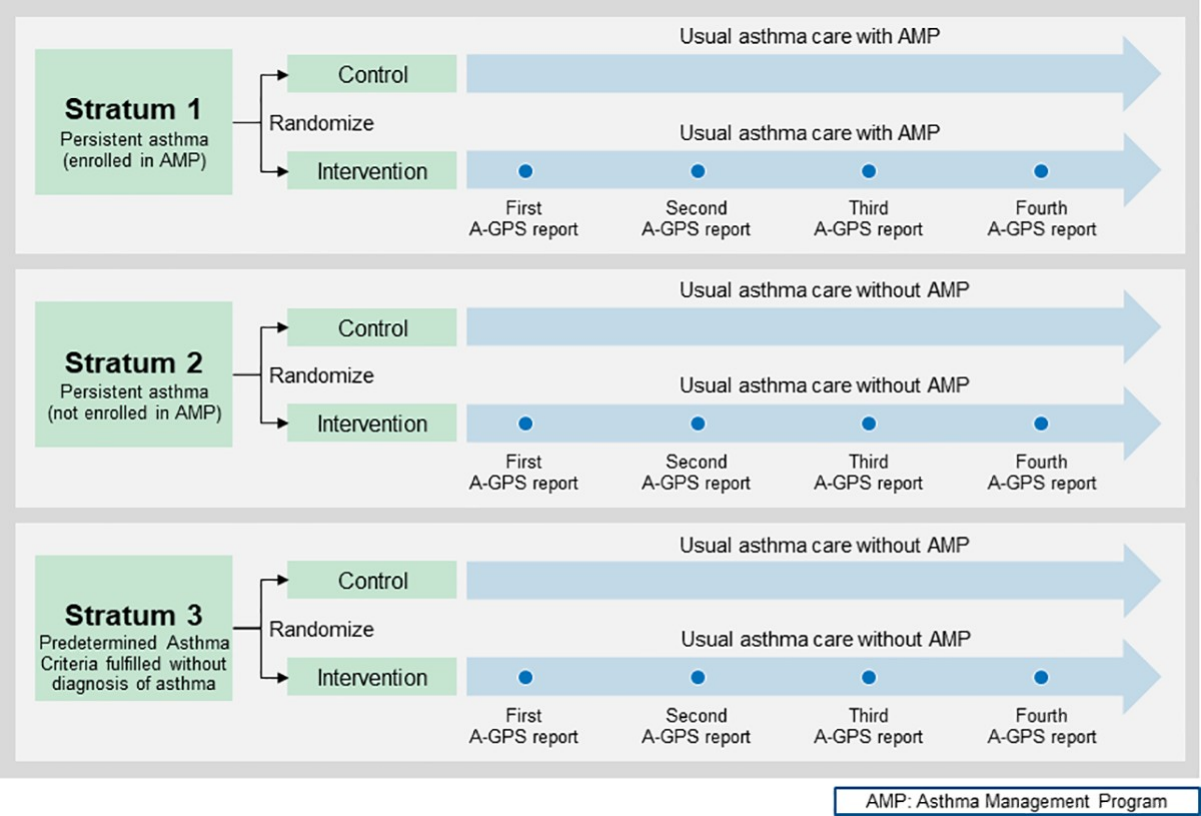
Study design for assessing the comparative effectiveness of A-GPS intervention via an RCT with stratified randomization (see S1 File. The Design and Implementation of Asthma-Guidance and Prediction System (A-GPS) for the definition for each stratum for details).

**Fig 2:**
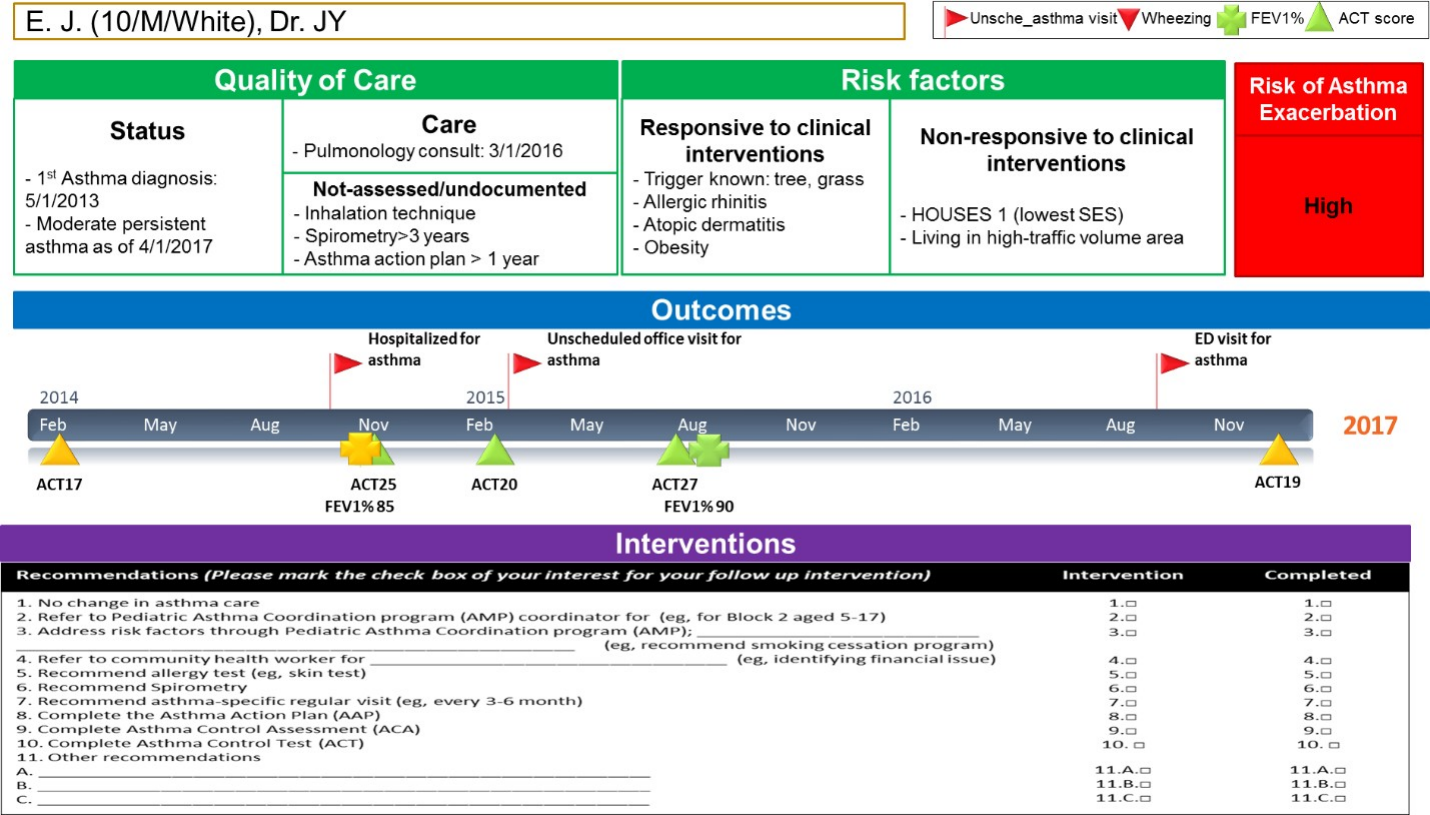
A-GPS report for individual patients provided to Primary Care Providers (PCPs) quarterly.

The study was designed as a single-center pragmatic RCT with a stratified randomization design for one year (Dec 13, 2016, to Dec 12, 2017) which assessed the effectiveness of A-GPS on asthma outcomes (Figs 1 and 2). This study was registered at the Clinical Trial Registration (NCT02865967). This study (IRB number:15–004435) was approved by the Mayo Clinic Institutional Review Board (IRB) on July 8, 2016.

Study subjects were stratified into three strata based on asthma severity, asthma care status, and asthma diagnosis.

Briefly, *Stratum 1* is comprised of children with persistent asthma (clinically defined by the Healthcare Effectiveness Data and Information Set (HEDIS) [16] and/or the National Asthma Education and Prevention Program (NAEPP) guideline [17] [see S1 File for details]), *enrolled* in an Asthma Management Program (AMP), a care coordination program for asthma. *Stratum 2* was a group of children with persistent asthma defined but *not* enrolled in AMP. Lastly, given the significant number (24%) of children without a diagnosis of asthma despite the recurrent asthma-like symptoms fulfilling asthma criteria (a delayed diagnosis of asthma) [18–21], *Stratum 3* included children with recurrent asthma-like symptoms who met Predetermined Asthma Criteria (PAC) in this pragmatic clinical trial which is summarized in S1 File. Predetermined Asthma Criteria developed by Drs. John Yunginger and Charles Reed, renown asthma researchers at Mayo Clinic is conceptually similar to the 2015 Canadian Thoracic and Canadian Pediatric Society asthma criteria (1. documented airflow obstruction, 2. Documented reversibility of airflow obstruction and 3. no evidence of an alternative diagnosis) based on patients’ EHRs but did not have a diagnosis of asthma yet [22, 23]. To our knowledge, PAC was the only existing predetermined criteria for asthma that determines asthma status and the index date of incident asthma retrospectively based on medical records using AI algorithm at the time of our study [24–26]. PAC was found to have high reliability, and extensive epidemiologic work for asthma has used PAC showing the excellent construct validity in identifying known risk factors for asthma and asthma-related adverse outcomes (e.g., serious and common infections) [27–39].

### Study population and setting

Olmsted County, southeastern Minnesota, is a virtually self-contained health care environment (only two health care systems provide clinical care to nearly all Olmsted County, MN residents), and 98% of residents authorize their medical records to be used for research [40]. According to U.S. census data in 2010, the age, sex, and ethnic characteristics of Olmsted County residents were similar to those of the state of Minnesota and the Upper Midwest [41]. However, Olmsted County has been becoming more diverse as indicated by the racial and ethnic characteristics of children enrolled in public schools (In 2019, 35.2% reported to be a non-white). The prevalence of asthma in a population of school-age children in our study setting, Olmsted County, MN, has been reported to be 17.6% [42]. Asthma is the most prevalent chronic illness with the third highest health care expenditures in children and adolescents in Olmsted County, MN [43]. Mayo Clinic Primary Care Pediatric Practices offer primary care service at four locations within Olmsted County, and this study was conducted in the primary care practice site (i.e., including teaching pediatric faculty, residents, and nurse practitioners).

### Study intervention

Subjects in each stratum were randomized to either A-GPS plus usual asthma care (intervention group) or usual asthma care only (control group) using computer-generated randomization and 1:1 assignment. The detailed development and implementation of A-GPS as intervention are described in S1 File The Design and Implementation of Asthma-Guidance and Prediction System (A-GPS). Also, we report all relevant data collected, and their definitions and data source in S1 File. Briefly, A- GPS report for each patient was provided to primary care providers (PCP) including pediatric residents and nurse practitioners at pre-determined dates *every three months* for PCP to review updated asthma status of each patient of theirs and determine whether the asthma management plan needed to be revised. Thus, PCP were *not blinded* to the intervention. A-GPS report included 1) summary of relevant information (quality of care, risk factors and outcomes) for asthma management (see 32 variables and their definitions and data sources listed in S1 File), 2) a machine learning (ML) algorithm (Bayesian classifier) forecasting the risk of AE in one year based on EHR data in the past 3 years (see S1 File for developing our ML algorithm for details), and 3) asthma management plans as shown in Fig 2. A-GPS report was generated by data mining tools including language processing (NLP) algorithms and ML algorithm based on preceding clinical encounters, patient-reported outcomes (eg, ACT score), and non-clinical data (eg, traffic volume and socioeconomic status) (see S1 File The Design and Implementation of Asthma-Guidance and Prediction System (A-GPS)).

After PCP reviewed the one-page updated A-GPS report every three months, they were offered multiple options to revise the current asthma management plans and informed the management plans (if they decided to change) to asthma care manager who carried out the revised management plans for each patient. The control group received usual asthma care without A-GPS report. As part of usual asthma care, we allowed parents to opt in and out of AMP, and AMP coordinators reached out and encouraged parents of persistent asthmatic children (e.g., Stratum 2) to be enrolled in AMP to offer care coordination for asthma to patients and their parents. Children and families were blinded to the study groups (i.e., intervention vs. control), but there was no deviation from usual asthma care described above. Inclusion criteria were as follows: children 1) under age 18 living in Olmsted County, 2) who had received medical care from pediatric primary care practice, Mayo Clinic, Rochester, between 2013 and 2016 and 3) research authorization using medical record for research. We enrolled the eligible children and invited their 42 PCP (teaching faculty and residents of pediatrics as well as nurse practitioners) to the study. All the PCP for eligible children participated in the study. Our study coordinators obtained and recorded the signed Health Insurance Portability and Accountability Act (HIPAA) form and the Mayo Clinic Institutional Review Board-approved verbal consent (See S1 File) from a parent or a guardian for study participation for their children given the nature of this minimal risk study.

### Endpoints

*1*. *Primary endpoint (effectiveness)*: Occurrence of AE within 1 year since the initiation of the study, defined by emergency department visits/hospitalization for asthma or unscheduled visits for asthma requiring oral corticosteroid [44].

2. *Secondary endpoints*:

1. Clinicians’ burden for reviewing and collecting clinical data from EHRs for making a clinical decision (efficiency): We surveyed 42 participating PCP asking how many minutes per each patient were spent reviewing A-GPS report for decision making (intervention group) and how many minutes per each patient they estimated would be needed to collect and review data listed in A-GPS without A-GPS report for their clinical decision making for asthma management (control group). We surveyed the participated PCP for the burden of EHRs review at the end of the study due to two main reasons: it was difficult for clinicians to accurately time for the duration of sporadic EHRs review for asthma care for patients in the control group (usual care) as they often review EHRs at multiple time points in a day or even longer whereas clinicians could report time spent for reviewing A-GPS report in the intervention group at the quarterly report time resulting in performance bias in estimating the burden for chart review; studies assessing clinician’s burden for reviewing EHRs for asthma management were often based on survey derived from their perceived burden instead of objective measurement of burden itself [2, 45]. We also asked two open-ended questions including “What parts of the current asthma care will be improved by the asthma-GPS report?” and “Which components of the asthma-GPS report can be improved upon?” (see their comments in S1 File).
2. Health care cost (efficiency): We defined health care cost in terms of direct medical expenses for each patient but did not include indirect costs such as patient’s out-of-pocket costs. Cost data were obtained from the Mayo Clinic Rochester Cost Data Warehouse (see S1 File for details). Detailed descriptions of this methodology have been published elsewhere [46].
3. Asthma control status (effectiveness): As an additional asthma outcome measure, quarterly asthma control status was measured by administering Asthma Control Test (ACT), Childhood ACT [1, 2, 47, 48], or Test for Respiratory and Asthma Control in Kids (TRACK) over the phone or via online [49, 50] depending on age (as ACT and Childhood ACT were validated for only children aged 4 years or above). The proportion of time (i.e., 4-time points for one year) when patients had well-controlled asthma (ACT or Childhood ACT>19 or TRACK>80) during the study period was determined.
4. Timeliness of asthma follow-up care after AE (efficiency): Any documented care for asthma either via clinic visit or by asthma care coordinator’s contact after AEs and time gap (in days) were retrieved as a measurement of asthma care quality.
5. Enrollment in an AMP (effectiveness): Stratum 2 was a group of children with persistent asthma (i.e., eligible for AMP as long as the age was 5 years or above) but not enrolled in AMP. We assessed how many children of the intervention vs. control groups participated in AMP during the study period because enrollment in AMP represented care quality improvement effort in addition to timeliness of asthma follow-up care after AE.

#### Covariates

We also collected pertinent variables on asthma outcomes, asthma care quality, and risk factors from EHRs which are described in S1 File.

#### Sample size estimation

Our hypothesis was that A-GPS intervention reduces the frequency of asthma exacerbation as the primary end point greater than usual care for asthma. According to the literature, average asthma intervention (e.g. care coordination) reduced asthma exacerbation as the primary end point defined by hospitalization and ED visits related to asthma by 59.2% and by 50.6%, respectively in a one-year time frame [51]. If we assume that the baseline prevalence rates of hospitalization and ED visits were 30.4% and 63.6% respectively and that the rate of asthma exacerbation in the control group rate decreased by 10% (relative reduction), with 80% power and a two-sided alpha level of .05, we would need approximately 100 subjects each for the intervention group and control group to detect the difference in reduction of the frequency of asthma exacerbation between intervention and control group (usual care).

#### Randomization and blinding

Once study subjects for each stratum were identified, children were assigned to intervention or comparison groups by computer (Excel, Microsoft) -generated sequential random numbers for all children in each stratum consecutively. Given the different nature of A-GPS and usual care and infeasibility of blinding, clinicians were not blinded to the assignment to intervention and control group (i.e., usual care).

### Statistical analysis

All analyses were performed using the intention to treat methods. For our primary outcome, we used a stratified logistic model to assess the association of A-GPS intervention with exacerbation during the study period. Additionally, to determine if the intervention reduced the proportion of subjects with AE during the trial (12 months) compared to the pre-trial time (12 months) more so than the control group, a logistic regression model predicting AE using the sandwich variance estimator was fit including the two times frames, group, and the interaction of group and time was examined. The median time to care team’s follow-up after AE was estimated using Kaplan Meier method and the estimated effect was given from Cox proportional hazards model with subjects with no follow up censored at the study end date. We utilized ANCOVA to compare the average proportion of time (ie, continuous variable) a subject was considered to have well-controlled asthma over their total follow-up period between two groups (intervention vs. control) while adjusting for age. A paired Wilcoxon signed-rank test (was used to assess clinicians’ time to review EHR with or without A-GPS to collect data listed in A-GPS. Difference-in-difference (DID) analysis (difference in incremental health care cost [from baseline to the end of study] between intervention and control group) was carried out to assess the different costs between intervention and control patients using a generalized linear modeling framework with cost having gamma distribution and logarithmic link. DID analysis was conducted through a linear regression of costs on time indicator (pre vs. post intervention), the intervention indicator and their interaction, so that the estimated coefficient interaction term provides treatment effect. The underlying standard error is generated through bootstrap sampling with 100 repetitions.

## Results

### Basic characteristics of study subjects

**Fig 3:**
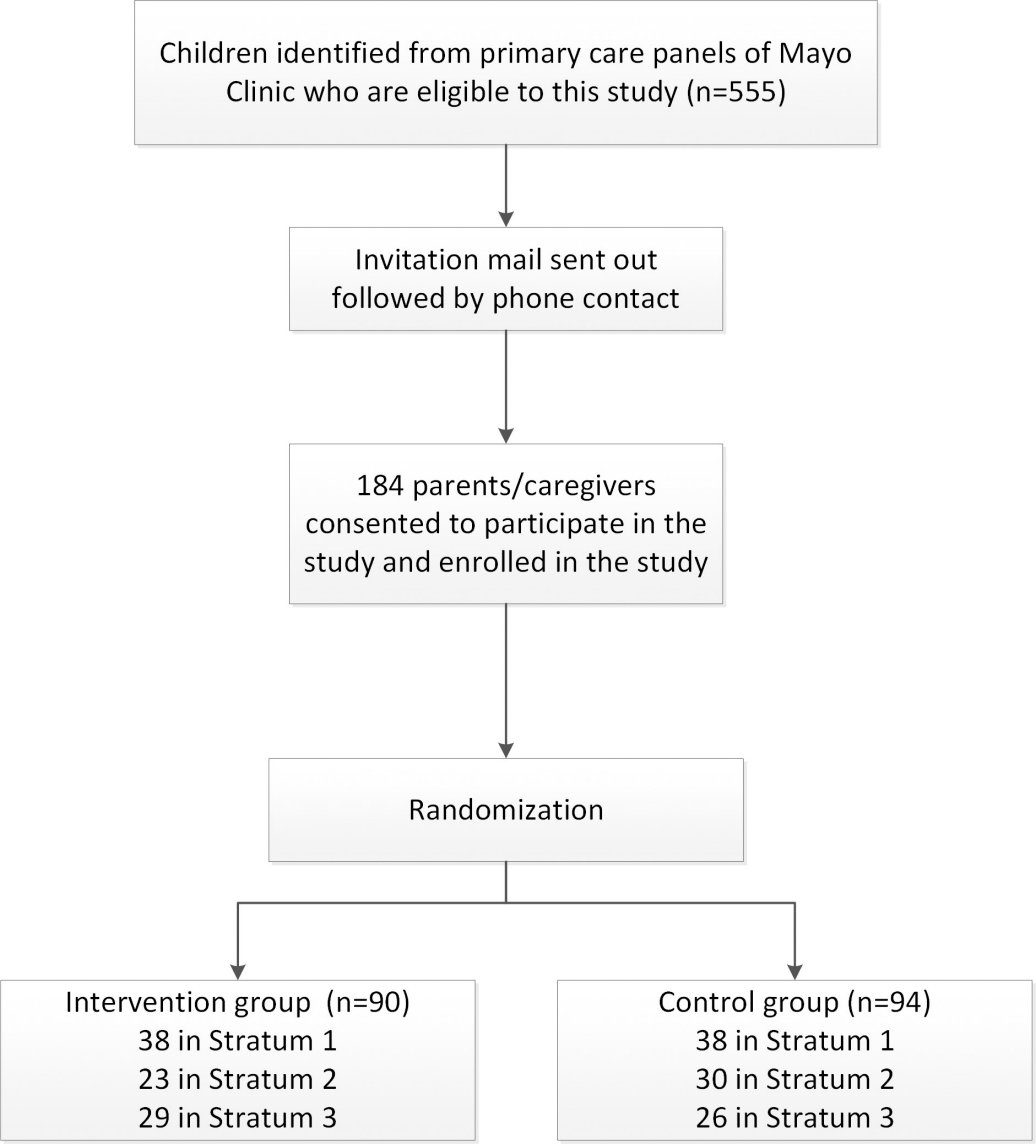
Consort flow diagram for subject enrollment and random allocation of subjects in each stratum to intervention vs control group (see Fig 1 for randomization, intervention and follow up).

Out of 555 who were invited to the study, 184 consented to participate in the study and were randomized (90 were assigned to the intervention group and 94 into the control group as shown in Fig 3).

The mean age at the time of enrollment was 9.0 years with 57% males and 72% Whites (Table 1). Overall, characteristics at baseline between the intervention group and the control group were not significantly different (Table 1).

**Table 1:** Baseline characteristics of study subjects between the intervention and the control.

	Total* (n = 184)	Intervention (n = 90)	Control (n = 94)
Age (year), median (IQR)	8.5 (5.2, 13.0)	9.3 (4.8, 13.3)	8.3 (5.4, 12.7)
Age subgroup, n (%)			
0–4 years	44 (24)	25 (28)	19 (20)
5–11 years	81 (44)	32 (36)	49 (52)
12–17 years	59 (32)	33 (37)	26 (28)
Male, n (%)	104 (57)	53 (59)	51 (54)
Stratum, n (%)			
Stratum 1	76 (42)	38 (42)	38 (40)
Stratum 2	53 (29)	23 (26)	30 (32)
Stratum 3	55 (30)	29 (32)	26 (28)
White race, n (%)	132 (72)	68 (76)	64 (68)
HOUSES^1^, n (%)			
Q1 (the lowest)	22 (13)	7 (8)	15 (17)
Q2	47 (28)	21 (25)	26 (30)
Q3	52 (30)	33 (39)	19 (22)
Q4 (the highest)	50 (29)	23 (27)	27 (31)
Living in high-traffic volume area, n (%)	29 (16)	15 (17)	14 (15)
Availability of patient online portal, n (%)	152 (83)	77 (86)	75 (80)
Family history of asthma, n (%)	90 (49)	50 (56)	40 (43)
History of eczema, n (%)	88 (48)	41 (46)	47 (50)
History of allergic rhinitis, n (%)	80 (44)	37 (41)	43 (46)
Seasonal influenza vaccine, n (%)	149 (81)	68 (76)	81 (86)
Discussion asthma at the general medical exam (GME)^2^^,^^3^, n/denominator (%)	50/91 (55)	26/47 (55)	24/44 (55)
Health care cost ($)^3^, median (IQR)	944 (492–1,939)	968 (538–1958)	932 (460–1,918)
History of asthma exacerbation^3^, n (%)	38 (21)	15 (17)	23 (25)
High-risk by prediction score^4^, n (%)	77 (42)	36 (40)	41 (44)
Primary care providers (PCP), n (%)			
Residents	55 (30)	26 (29)	29 (31)
Other PCP	129 (70)	64 (71)	65 (69)

^1^ HOUSES: An individual-level socioeconomic status measures based on real property data. The number of missing in the intervention and the control group were 6 and 7, respectively

^2^ Among only those who had GME (47 for the intervention and 44 for the control group)

^3^ During timeframe of 12 months prior to clinical trial

^4^ Prediction score (high vs. low) of the likelihood of having AE within future 1 year based on EHR data in the past 3 years

### Primary endpoint

**Fig 4:**
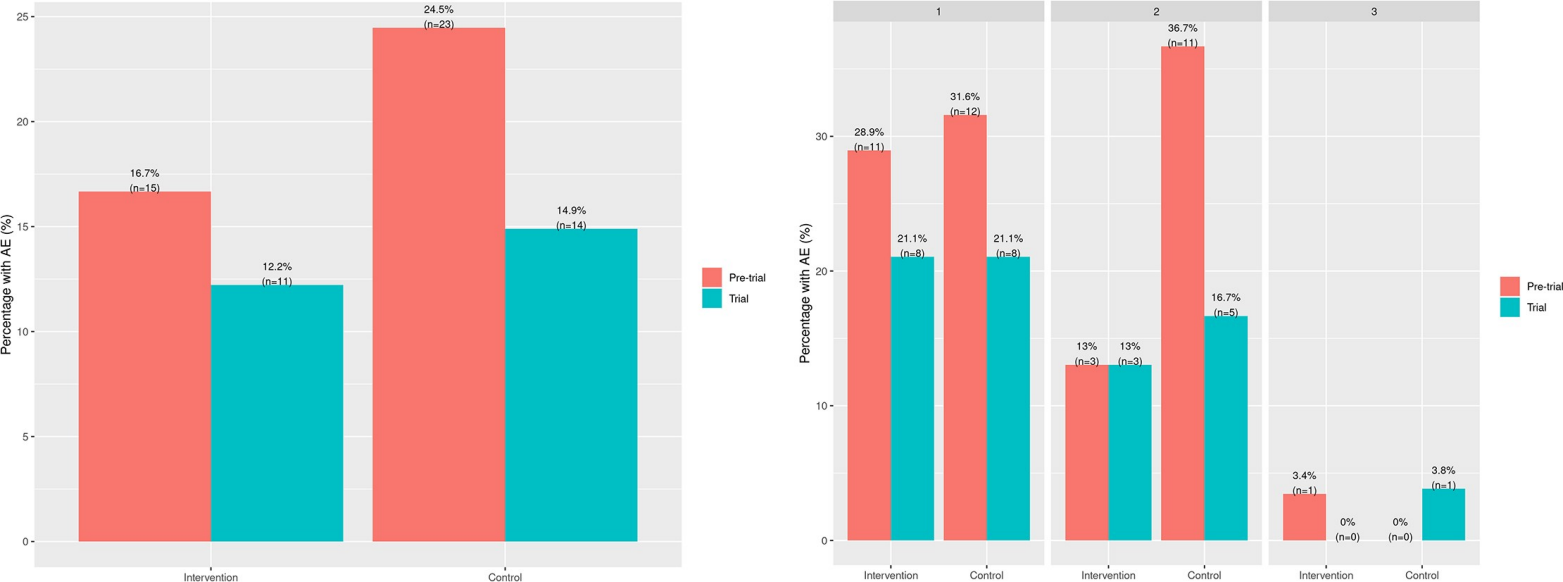
Comparisons of the frequency of asthma exacerbation (the primary endpoint) between the intervention and control group (Fig 4A for overall intervention and control group and Fig 4B for individual strata) during the 12 months prior to the trial and the 12-month trial. **A.** Comparison between overall intervention and control group. **B.** Comparison between intervention and control group at pre-trial and trial by stratum.

Because of the guidance that A-GPS provided, PCPs took a significantly higher number of actions (interventions) for intervention group (eg, 18 referrals to AMP (vs. 7 in control group), 5 referrals to community health workers (vs. 0), requests for 8 skin tests (vs. 5), 23 spirometry (vs, 12), 33 asthma-specific regular visits (vs. 4), and 28 ACT update (vs. 7)), of which were executed by asthma care team, compared to control group. The main results on the comparison in primary outcomes between the intervention and control groups are summarized in Fig 4 and Table 2.

**Table 2:** Primary and secondary outcomes in intervention and control groups.

	Intervention (n = 90)	Control (n = 94)	P-value
Primary outcome			
Asthma exacerbation, n (%)	11 (12%)	14 (15%)	0.60
Secondary outcomes**			
Clinician’s time in minutes taken to make clinical decision, median (IQR)	3.5 (2–5) min	11.3 (6.3–15) min	<0.001
Health care cost ($), mean (95% CI)**			
Pre-intervention predicted cost	$2474 ($1540, $3409)	$1721 ($1085, $2357)	
Post-intervention predicted cost	$1438 ($895, $1981)	$1800 ($1135, $2466)	
Difference	-$1036 (-$2177, $44)	$80 (-$841, $1000)	0.12
Percentage of the duration of well-controlled asthma by quarterly ACT or TRACK score (%), median (IQR)	100 (75 to 10)	100 (100 to 100)	0.56
Timeliness for follow-up care after asthma exacerbation (days)^, median (IQR)	7 (2 to 27)	28 (6 to 48)	0.10

^Using Kapan Meier method among those with asthma exacerbation (11 for the Intervention and 14 for the control group)

**Clinician’s time for making clinical decision related to asthma for the intervention vs. the control group was compared, while clinicians themselves were not randomized.

During the study period, overall, 14% (n = 25) of the subjects had at least one AE whereas 21% (n = 38) developed AE during one year prior to the trial. The proportion of children with AE in both groups decreased from the baseline (P = 0.042), specifically 17% → 12% in the intervention vs. 25% → 15% in the control group (Fig 4A). However, there was no difference in AE frequency between the two groups (12% for the intervention group vs. 15% for the control group, block stratified Odds Ratio: 0.82; 95%CI 0.34–1.96; P = 0.66) during the study period. Outcome comparisons among three strata as *a post-hoc* analysis done showed no statistical differences in the primary endpoints by each stratum (Fig 4B): control groups had a reduction in AE in strata 1 and 2 regardless of the availability of AMP and while there was no AE event in the intervention group, 4% of the control group had AE events in stratum 3.

### Secondary endpoints

The results on secondary outcomes are summarized in Table 2. Forty-two primary care providers participated in this trial and 28 (67%) completed surveys. A-GPS significantly reduced clinicians’ burden for chart review to make clinical decision for asthma management by 67%, with an estimated median time to review patient’s medical records of 3.5 min (IQR: 2–5) with A-GPS intervention vs. 11.3 min (IQR: 6.3–15) without A-GPS (P<0.001). Average decrease within a person with A-GPS (vs. without A-GPS) was 7.3 min. This pattern was similarly found in residents (an average decrease of 7 min) and other PCP (8 min). The median overall self-reported satisfaction analog score for A-GPS was 7 (1: lowest, 10: highest) after the study was completed. Other comments are described in S1 File.

Mean health care costs with 95%CI of children during the trial (compared to before the trial) in the intervention group were lower than those in the control group (-$1,036 [-$2177, $44] for the intervention group vs. +$80 [-$841, $1000] for the control group; Table 2), though there was no significant difference (p = 0.12). These heath care costs did not take into account clinician’s time to review EHRs.

There was no difference in the proportion of duration when patients had well-controlled asthma during the study period between the intervention and the control groups as shown in Table 2. However, asthma control status at baseline was strongly associated with the asthma control status over follow up even after adjusting for age (age-adjusted difference = 0.26; P<0.001).

Among those who experienced AE during the study period (n = 25), those in the intervention group had timelier follow up by the clinical care team compared to those in the control group but no significant difference was found (HR = 1.93; 95% CI: 0.82–1.45, P = 0.10). Among children in Stratum 2, a total of 8 children in the intervention group were enrolled in AMP whereas none of the control group participated in AMP during the study period.

### Adverse events

There were 0 serious adverse events resulting in death or breach of confidentiality in both intervention and control groups.

## Discussion

To our knowledge, this exploratory pragmatic trial is the first RCT that assessed the comparative effectiveness of an AI-assisted CDS leveraging EHRs for asthma management in reducing AE. While A-GPS showed similar reduction in AE events to usual care, A-GPS reduced clinicians’ self-reported burden for reviewing EHRs by 67% compared to the control group and provided more actionable guidance for asthma management to PCPs in a way better addressing the needs of patients, compared to control group (usual care).

As both intervention and control groups showed a reduction of AE from the baseline, it reduced the comparative effectiveness of the intervention in our study. It is, however, difficult to disentangle the effect of A-GPS from other effects (e.g., ripple effect of intervention, effect of AMP or secular trend). We do not think these observations were due to the effect of AMP, a care coordination program for children with persistent asthma which was available only to *stratum 1 because* our study results showed a similar reduction of AE in the control group in *stratum 2* (children with persistent asthma *who were not enrolled in AMP*). Also, secular trend of improvement of AE is unlikely as we observed reduction of AE in the intervention group but increase of AE in the control group in stratum 3, although it was not statistically significant. Thus, we postulate that as clinicians were not blinded to the study assignment, the intervention with A-GPS might reduce AE in both intervention and control groups as *a ripple effect* (beneficial effects of interventions in untreated control groups). This effect has been widely recognized in observational studies and clinical trials [52–54]. However, given the limited RCT for AI or HIT-assisted interventions, the degree and nature of a ripple effect in adoption and implementation of HIT are poorly understood in the AI or HIT literature, although empirical observations for a ripple effect in HIT have been reported [55]. A few noteworthy aspects need to be considered for interpreting our study results. In *stratum 2*, there were only 3 patients out of 23 children (13%) in the intervention group who had a history of AE in the prior year before the study, compared to 11 of 30 children (37%) in the control group, suggesting a potential imbalance of covariates despite randomization which might impact the results. In addition, under usual asthma care practice, our study permitted study participants to opt in and out of AMP (depending on asthma control status assessed by clinicians and parental choice as long as the age was≥ 5 years) during the study period. As all 8 children, who were enrolled in AMP during the study period, were the intervention group and they were more likely to be children with poorly controlled asthma, this might be one of reasons which could reduce the effectiveness of the intervention in stratum 2.

A-GPS intervention might significantly reduce clinicians’ burden (estimated time for chart review and clinical decision making) by 67%, compared to the control group (3.5 minutes vs. 11.3 minutes, respectively). This benefit remained similar across PCP including pediatric residents. While the average reduced time for EHRs review (7.3 min) seems to be small, given the reported average duration for clinical review for EHRs (14.8 to 17 min) in the US and elsewhere [5], this effect size for reducing clinician’s burden for EHRs review for asthma management might represent a major benefit of A-GPS intervention achieving efficient and effective asthma management. Importantly, while A-GPS reduced clinicians’ burden for reviewing EHRs, it provided significantly more actionable guidance for asthma management to PCPs in a way better addressing the needs of patients, compared to control group (usual care). We believe this is a key benefit of A-GPS in asthma care.

In the context of health care costs, A-GPS intervention reduced direct health care costs from those for the prior year, compared to the control group but no significant difference was found (-$1,036 for the intervention group vs. +$80 for the control group, p = 0.12). However, we did not include the reduction of clinician’s time and effort in the cost-benefit analysis because it is not captured in billing data, which was used for estimating costs. Also, although statistically not significant, the intervention group had a timelier follow up after AE, compared to the control group (7 vs. 28 days, P = 0.10), given the importance of follow-up care for AE on asthma management, this timelier follow up in the intervention group in this study could represent an important benefit of A-GPS. Also, as discussed above, children with persistent asthma in the intervention group were significantly more likely to participate in AMP, compared to the control group. This care quality improvement might reflect that A-GPS helps PCP and care team better capture the unmet needs of families and improve access to AMP. Given our study as an under-powered exploratory study and the potential benefits, our study findings need to be assessed with studies with larger sample size.

Our study has several limitations. First, this study was a single-center single-blinded pragmatic trial, which limits its generalizability to other study settings and makes it difficult to minimize performance bias, which potentially resulted in a ripple effect as discussed above. Second, our study as an exploratory pragmatic trial fully integrated in a real-world primary care practice setting, was limited by a small sample size to be handled by Asthma Management Program (AMP) care coordinators of clinical practice and relatively lower AE incidence both in previous year and during the clinical trial despite our effort to enroll as many children with a history of AE as possible. Third, our study did not include lung function measures or medications in defining persistent asthma and study outcomes but included only clinical outcomes as a pragmatic trial. Especially, identification of Stratum 3 was based on PAC which has its own limitations in ascertaining asthma status in children. Fourth, the intervention was not synchronized with a clinical visit for asthma but prescheduled quarterly report for intervention which might have reduced the effectiveness of the intervention. Lastly, the population of Olmsted County, Minnesota is predominantly white (90%) and Scandinavian in ancestry, which may limit the generalizability of study results to other racial/ethnic groups. Also, our study has a few important strengths. First, our study included the existing AI tools in A-GPS report such as NLP algorithms for asthma ascertainment (NLP algorithms for PAC [24–26] and Asthma Predictive Index [56]) who showed promising performance in ascertaining asthma status using EHRs on a large scale. Also, we incorporated clinically unavailable data such as individual-level SES as measured by validated HOUSES and traffic volume data know to trigger asthma exacerbation [57, 58] which provide clinicians with contextual information in asthma management. None of participating PCP and only 7% of subjects were censored (loss of follow up) before the completion of the study. Our study setting has an epidemiological advantage of being self-contained health care environment which is likely to capture all asthma-related events during the study period.

In conclusion, while A-GPS-based intervention showed similar reduction in AE events to usual care, it might have a potential to reduce clinicians’ burden for EHRs review resulting in more efficient asthma management while providing more actional guidance for asthma management. Cluster randomized trials with a larger sample size are needed to further assess the effectiveness and external validity of A-GPS.

## Supporting information

S1 File Supplement. (DOCX)

S2 File Consort checklist. (PDF)

S3 File IRB protocol. (PDF)

## Abbreviations

A-GPS: Asthma-Guidance and Predictive System
AI: Artificial Intelligence
AMP: Asthma Management Program
API: Asthma Predictive Index
CDS: Clinical Decision Support
EHR: Electronic Health Records
HIT: Health Information Technology
ML: Machine Learning
NAEPP: National Asthma Education and Prevention Program
NLP: Natural Language Processing
PAC: Predetermined Asthma Criteria
PCP: Primary Care Providers
RCT: Randomized Clinical Trial
