# Supplementary material for: Artificial intelligence-assisted clinical decision support for childhood asthma management: A randomized clinical trial
Source: PLoS One. 2021 Aug 2;16(8):e0255261. doi: 10.1371/journal.pone.0255261 (PMC8328289; doi:10.1371/journal.pone.0255261)
Supplement: S1 File — (DOCX) [file pone.0255261.s001.docx]

**Contents of Supplement**

- The Design and Implementation of Asthma-Guidance and Prediction System (A-GPS)
- Health care cost measurement
- Persistent asthma definition by the Healthcare Effectiveness Data and Information Set (HEDIS)^27^ and by risk of the National Asthma Education and Prevention Program (NAEPP) guideline^1,^
- Two Asthma Criteria used for this study
- Variables used for A-GPS and their definitions.
- Comments from survey to primary care providers
- References for Supplement
- Verbal Consent Script

***The Design and Implementation of Asthma-Guidance and Prediction System (A-GPS)***

A-GPS was designed, developed, implemented, and assessed in a primary care practice setting for one year and tested via a randomized clinical trial (RCT).

***1) Designing phase****:* We held advisory group meetings consisting of primary care providers and allergy specialists to discuss the design of A-GPS based on NAEPP 2007 guidelines^1^. We had survey and focus group interviews with them to identify and define the data that would be helpful to the PCP for asthma management through online REDCap (NIH/NCATS UL1 TR000445). We listed 32 asthma-related variables (e.g., care quality, risk factors, and outcomes) suggested by the advisory group and prioritized them based on the survey results and current technical feasibility as summarized in S3 Table in Supplement 2.

***2) Development phase:*** A-GPS consisted of; 1) an outline to guide asthma care specific to patient: this module had retrieved and summarized structured and unstructured data on asthma care quality, risk factors including individual-level socioeconomic status (SES) and environmental factors, and asthma outcomes extracted from individual patient’s EHRs using NLP algorithms for unstructured data (see S3 Table), and 2) Prospective prediction of AE: using the retrospectively collected data, we developed (trained) and validated predictive analytics machine learning model for acute exacerbation (see Predictive analytics for AE below) which was used in our RCT prospectively.

Computerized A-GPS was developed for this RCT which retrieved, summarized, and reported relevant information for asthma management including 1) quality of care measures, 2) risk factors for AE including individual-level SES and geospatial measurement of environmental risk, and 3) asthma outcome monitoring for unstructured data and structured data as described below (see S3 Table).

Quality of care: The variables on quality of asthma care that were reported to PCP are listed in S3 Table (e.g., allergens/triggers, laboratory test, and Asthma Action Plan) based on NAEPP guidelies.^1^ For patients with undiagnosed asthma despite their recurrent asthma-like symptoms, A-GPS reported PAC (S2-1 Table^2^ which is similar to asthma criteria by the Canadian Thoracic Society and Pediatric Society^3^) positivity with an index date which is the earliest date when one met the criteria. This was generated by validated NLP algorithms for PAC^4,5^ and the status of Asthma Predictive Index (API; S2-2 Table^6^) by validated NLP algorithm for API^7^ to PCP. For the patients with persistent asthma (i.e., Stratum 1 and 2), A-GPS reported the first date of asthma diagnosis, current level of asthma severity, and history of specialty care for asthma in the past 3 years. Among these variables, undocumented or unavailable tests were marked under the section of “Not-assessed/Undocumented” to prompt primary care providers to consider.

Risk factors for asthma: As clinicians spend a great deal of time obtaining a relevant history focusing on risk factors for asthma from parents and patients and document those in medical records, A-GPS report provided asthma-related risk factors to support the PCP’s further decision for children with undiagnosed asthma despite recurrent asthma symptoms. For the persistent asthmatics, asthma-related risk factors were categorized into “risk factors responsive to clinical interventions (modifiable)” and “risk factors non-responsive to clinical interventions (non-modifiable)” to aid the primary care providers to consider preemptive care or education to mitigate exposure to modifiable risk factors for AE^8-11^, although this categorization may be arguable. We also included two non-clinical variables which were not routinely available in EHRs but were known to be associated with asthma outcomes; 1) Social determinants of health such as validated individual HOUsing-based SocioEconomic Status (HOUSES) index which is known to be associated with asthma incidence and control status^12,13^, and geospatial measurement of environmental risk factors such as living nearby high traffic volume as a marker of exposure to air pollution^14-18^. Air pollution in road traffic is a serious health hazard for asthma patients^17-20^, and we estimated heavy commercial vehicle average daily traffic (HCADT) in Olmsted County based on data obtained from Minnesota Department of Transportation and City of Rochester Public Works Department. The correlation between estimated HCADT and actual diesel vehicle volume in a random-sampled 6 locations in this community (Spearman’s rho) was 0.9 (p=0.037).

Assessment of asthma outcomes: The visual summary of monitoring of asthma outcomes for three years were generated in the middle of the individual report with chronological dates (Figure 2) for helping primary care providers to easily interpret recent status of the children. Asthma exacerbation (which is defined by an emergency room (ED) visit, hospitalization, or outpatient visit requiring systemic corticosteroids for asthma^21^), pulmonary function test (FEV1), methacholine challenge test, and Asthma Control Test^22,23^ were included. For subjects who met PAC without physician-diagnosed asthma, recurrent asthma symptoms for meeting PAC and their incidence dates were included in the individual report. Validity of history of AE captured by A-GPS (e.g., NLP-empowered algorithms using the Mayo Clinic's Advanced Cohort Explorer [ACE], a structured and non-structured (e.g., free text) data extraction tool for clinical data repository maintained by the Unified Data Platform) for two years (one year before and after the first intervention date by A-GPS) showed 88% (28/32) of sensitivity and 99% (3089/3091) of specificity with the manual review of medical records as the reference at the note level.

Asthma management plans: As shown in Figure 2 (bottom part), to ease revision of the current asthma management plans by PCP, A-GPS report offered 10 different designated asthma management plans and additional plans to be considered after reviewing the summary report of A-GPS by PCP. The list of asthma management plan options was developed and updated per PCP’s request during the study period.

Predictive analytics for AE: To develop a machine learning model for predicting acute exacerbation within 1 year, we extracted 29 candidates of variables based on the literature^24,25^ including sociodemographic, risk factors, and asthma outcomes over the past three years (S3 Table). While eosinophil count and FeNO were initially considered for model development, we included only total IgE count considering its collinearity with eosinophil and FeNO as well as limited availability of FeNO and eosinophil counts in EHRs. Using a convenient sample of 590 subjects which was part of Mayo Clinic pediatric practice panel, 300 subjects were set aside as the training set, and 290 subjects were set aside as the independent validation set. Then, we employed a Naïve Bayes machine learning approach to identify predictors for AE and investigate the collective predictive capability of the predictors for future AE.^26^ The final prediction model developed based on Naïve Bayes machine learning approach with five most contributable variables (i.e., previous exacerbation, symptom, hospital visit, rescuer medication, and controller medication) was used. We updated the providers with the prediction score using the 5 variables every three months for PCP (high vs. low; based on a cutoff for Naïve Bayes machine learning model derived from receiver operating characteristic (ROC) curve). Performance of machine learning model using those five variables was comparable without significant loss of performance to all 29 variables. (S3 Table) ROC Areas Under Curve were 0.74 and 0.78 on the development and validation cohort, respectively.

***3) Implementation phase:*** The eligible participants of this study were randomized to the intervention (A-GPS with usual asthma care) and the control (only usual asthma care) groups. A-GPS intervention included an individual report generated by the A-GPS (Figure 2) was provided to the PCP at a quarterly meeting as a AI-assisted CDS tool for asthma management, and then PCP revised asthma management plan supported by the A-GPS report with a nurse care coordinator of asthma management program or other nurse care team.

*Stratum 1:* children with persistent asthma enrolled in the Asthma Management Program [AMP] (a care coordination program for children with persistent asthma); *Stratum 2:* children with persistent asthma *not enrolled* in asthma management program (e.g., parents opted out of AMP); persistent asthma for Stratum 1 and 2^1,27^ (see S1 Table in Supplement 2); *Stratum 3:* children with recurrent asthma-like symptoms per patient’s EHR who met Predetermined Asthma Criteria (PAC) but did not have a diagnosis of asthma in EHR. NLP algorithm for PAC and Asthma Predictive Index (API) (see S2 Table in Supplement 2)^2^ was applied to EHR of intervention group (i.e., NLP algorithms for asthma criteria^4,5^) to help PCP ascertain asthma status and diagnose with asthma.

For Stratum 1, nurse care coordinator executed the changed care plan per PCP’s recommendations and provided patients with any care coordination for asthma care necessary as care coordination typically takes a great deal of clinician’s time (e.g., addressing social determinants of health in delivery of asthma care). Thus, nurse care coordinator of asthma management program played an integral role in asthma care in this study setting.

Intervention

Every three months, an individual report was generated by A-GPS and provided to PCP. After reviewing the one page A-GPS report as shown in Figure 2, PCP revised asthma management plans and marked in the checkbox for management plans listed in A-GPS report they want to change or add for asthma management under “Recommendations”. The detailed development and implementation of A-GPS as intervention are described in *the Design and Implementation of Asthma-Guidance and Prediction System* in Supplement 1 above.

It consisted of the following three components:

*1. Guidance system for summarizing all relevant clinical information for asthma management:* Relevant information for asthma management based on NAEPP guidelines and inputs from the advisory meetings were retrieved, summarized, and reported by NLP algorithms and text mining program. The definition, data source and data mining tools for the 32 variables used are described in S3 Table in Supplement 2.

*2. Predictive analytics for forecasting the AE in the future*: The detailed description for training and testing machine learning (ML) algorithm we used for predicting AE is described under *Predictive analytics for AE above*.

3. *Asthma management plans:* As shown in Figure 2, A-GPS report offered 10 different designated asthma management plans and additional plans to be considered after reviewing the summary report of A-GPS by PCP as described above.

*Control*

The control group received usual asthma care without A-GPS report. As this study was a single blinded RCT (patient/parent only), we were not able to conceal the assignment of study subjects from the participating PCP. As part of usual asthma care, we allowed parents to opt in and out of AMP, although AMP reached out and encouraged parents of high-risk persistent asthmatic children to be enrolled in AMP to address health care access to AMP.

***Health care costs***

The Mayo Clinic Rochester Cost Data Warehouse provides a standardized cost for each billed service. Professional services are valued based on year-specific Medicare reimbursement rates based on CPT4 billing codes. Hospital facility costs are valued using billed charges multiplied by department level cost-to-charge ratios provided by the annual Medicare hospital cost reports. All costs were inflated to 2017 US Dollars using the Gross Domestic Product Implicit Price Deflator. Detailed descriptions of this methodology have been published elsewhere.^28^ In addition, it did not include the cost from the clinician’s differential time for collecting and reviewing clinical data from EHRs which was measured separately as described above.

| **S1 Table. Persistent asthma definition by the Healthcare Effectiveness Data and Information Set (HEDIS)^27^ and by risk of the National Asthma Education and Prevention Program (NAEPP) guideline^1^** |
| --- |
| **A. HEDIS**  1) At least one ED visit, with a principal diagnosis of asthma  2) At least one acute inpatient encounter, with a principal diagnosis of asthma  3) At least four outpatient visits or observation visits on different dates of service, with any diagnosis of asthma AND at least two asthma medication dispensing events.  4) At least four asthma medication dispensing events  **B. NAEPP**  1) 0-4 years: ≥2 exacerbations in 6 months requiring oral systemic corticosteroids, or ≥4 wheezing episodes/1 year lasting >1 day AND risk factors for persistent asthma  2) ≥5 years: ≥2 exacerbations in 1 year requiring oral systemic corticosteroids  * In addition to HEDIS and NAEPP, if patient had physician diagnosis of persistent asthma in the clinical note within the past 12 months, or physician diagnosis of asthma in the clinical note AND on controller medication in the past 12 months, we included them for either Stratum 1 or 2. |
|  |

| **S2 Table: Two Asthma Criteria used for this study** | |
| --- | --- |
| **2-1. Predetermined Asthma Criteria (PAC)** | |
| Patients were considered to have *definite* asthma if a physician had made a diagnosis of asthma and/or if each of the following three conditions were present, and they were considered to have *probable* asthma if only the first two conditions were present:   1. History of cough with wheezing, and/or dyspnea, OR history of cough and/or dyspnea plus wheezing on examination, 2. Substantial variability in symptoms from time to time or periods of weeks or more when symptoms were absent, and 3. Two or more of the following:  - Sleep disturbance by nocturnal cough and wheeze - Nonsmoker (14 years or older) - Nasal polyps - Blood eosinophilia higher than 300/uL - Positive wheal and flare skin tests OR elevated serum IgE - History of hay fever or infantile eczema OR cough, dyspnea, and wheezing regularly on exposure to an antigen - Pulmonary function tests showing one FEV_1_ or FVC less than 70% predicted and another with at least 20% improvement to an FEV_1_ of higher than70% predicted OR methacholine challenge test showing 20% or greater decrease in FEV_1_ - Favorable clinical response to bronchodilator   Patients were excluded from our previous study if any of these conditions were present:   - Pulmonary function tests that showed FEV_1_ to be consistently below 50% predicted or diminished diffusion capacity - Tracheobronchial foreign body at or about the incidence date - Hypogammaglobulinemia (IgG less than 2.0 mg/mL) or other immunodeficiency disorder - Wheezing occurring only in response to anesthesia or medications - Bullous emphysema or pulmonary fibrosis on chest radiograph - PiZZ alpha_1_-antitrypsin - Cystic fibrosis - Other major chest disease such as juvenile kyphoscoliosis or bronchiectasis   *FVC forced vital capacity; FEV1, forced expiratory volume in 1 sec.* | |
| **2-2. Asthma Predictive Index (API)** | |
| **Major Criteria** | **Minor Criteria** |
| 1. Physician diagnosis of asthma for parents 2. Physician diagnosis of eczema for patient | 1. Physician diagnosis of allergic rhinitis for patient  2. Wheezing apart from colds  3. Eosinophilia (≥ 4%) |
| * Asthma is determined by frequent wheezing episodes (two or more) plus at least one of two major criteria or two of three minor criteria | |

| **S3 Table. Variables used for A-GPS and their definitions.** | | |  |  |
| --- | --- | --- | --- | --- |
|  | | **Definition** | **Data source** | **Retrieval method** |
| **ASTHMA OUTCOMES** | | | | |
| Emergency room/Hospitalization/Oral corticosteroid use with asthma-related events | | Asthma-related events including asthma, reactive airway disease, bronchiolitis, bronchospasm, wheezing, dyspnea, shortness of breath, allergic bronchitis under physician diagnosis section | EHRs  (clinical notes) | ACE |
| Unscheduled outpatient visit with asthma-related events | |  | EHRs (clinical notes) | ACE |
| Wheezing episodes | | Wheezing documented from physical exam or history-taking | EHRs (clinical notes) | NLP |
| Score of Asthma Control Test (ACT) | | ACT score as a continuous variable | EHRs (structured codes) | ACE |
| Pulmonary function test (FEV1) | | The latest result of FEV1 within the past 5 years | EHRs (structured codes) | ACE |
| Methacholine challenge test | | The result of methacholine challenge test ever | EHRs (structured codes) | ACE |
| **ASTHMA CARE QUALITY** | | | | |
| Predetermined Asthma Criteria (PAC) date | | Date when PAC was first fulfilled (asthma onset date) | EHRs (clinical notes) | NLP |
| Asthma Predictive Index (API) date | | Date when API was first fulfilled | EHRs (clinical notes) | NLP |
| Physician diagnosis of asthma | | Date of first physician diagnosis | EHRs (clinical notes) | NLP |
| Asthma severity | | The latest physician assessed asthma severity | EHRs (clinical notes) | ACE |
| History of asthma care by specialists | | The latest in-person clinical visit to allergist or pulmonologist | EHRs (structured codes) | ACE |
| Status of allergens/triggers/smoking available | | Status of allergens or triggers by self-report | EHRs (structured codes) | ACE |
| Education for inhaler technique | | Inhaler technique taught or observed | EHRs (clinical notes) | NLP |
| Asthma management at well-child visit | | Asthma discussed and documented during general medical exam in the past 1 year | EHRs (clinical notes) | ACE |
| History of pulmonary function test | | Status of pulmonary function tests performed within the past 3 years | EHRs (structured codes) | ACE |
| History of allergic skin tests or total/allergen specific immunoglobulin E | | Status of skin test or blood test for inhaler allergens performed ever | EHRs (structured codes) | ACE |
| History of peripheral eosinophil count | | Status of peripheral eosinophil count from blood test performed ever | EHRs (structured codes) | ACE |
| History of Asthma Action Plan Up-To-Date status | | Status of Asthma Action Plan available within the past 1 year | EHRs (structured codes) | ACE |
| **MODIFIABLE RISK FACTORS** | | | | |
| Known Triggers of Allergen | | Allergens or triggers documented by self-report, skin test, and/or blood test | EHRs (structured codes) | ACE |
| Smoking exposure | | Smoking status documented by self-report | EHRs (clinical notes) | ACE |
| Asthma medication adherence | | Asthma medication adherence by self-report  (only available for Stratum 1) | EHRs (clinical notes) | ACE |
| Seasonal flu vaccine | | Status of seasonal flu vaccination in the same as or year prior to the study period | EHRs (structured codes) | ACE |
| History of September epidemic for asthma | | September epidemic defined by emergency room visit, hospitalization, and/or oral corticosteroid use for asthma between Aug and Sep in the past 3 years | EHRs (clinical notes) | ACE |
| History of allergic rhinitis | | Diagnosis of allergic rhinitis documented by care provider in the past 3 years | EHRs (clinical notes) | ACE |
| History of depression | | Diagnosis of depression documented by care provider in the past 3 years | EHRs (clinical notes) | ACE |
| History of obesity | | Diagnosis of obesity documented by care provider in the past 3 years | EHRs (clinical notes) | ACE |
| **UNMODIFIABLE RISK FACTORS** | | | | |
| HOUSES index | | Individual level socioeconomic status | Real property data | HOUSES Program |
| Living in a ‘hotspot’ for high-traffic volume | | Hotspot defined by geospatial analysis for living nearby high traffic volume as a marker of exposure to air pollution by heavy commercial vehicle average daily traffic | Minnesota Department of Transportation and City of Rochester Public Works Department | HOUSES Program |
| Family history of asthma | | 1^st^ degree family history of asthma by self-report | EHRs (structured codes) | ACE |
| History of atopic dermatitis | | Diagnosis of atopic dermatitis (eczema) documented by care provider in the past 3 years | EHRs (clinical notes) | ACE |
| Increased serum total IgE | | Increased serum total IgE compared to age-specific reference range | EHRs (structured codes) | ACE |
| Increased serum eosinophil count | | Increased serum total eosinophil counts compared to age-specific reference range | EHRs (structured codes) | ACE |
| *ACE (Advanced Cohort Explorer, clinical data repository maintained by the Unified Data Platform including text search functionality); ** NLP (Natural Language Processing); *** HOUSES Program: Mayo Research Core program which provides HOUSES (HOUsing-based SocioEconomi Status) index and geospatial analysis service. | | |  |  |

**Comments from survey to primary care providers**

1. “A-GPS can be a tool to be implemented in specialty practice (eg, when a patient is referred to a specialty practice, A-GPS report can be accompanied with the order to reduce specialists’ effort and time for chart review)”,
2. “It would be more beneficial if A-GPS report is provided as a clinical decision tool integrated in EMRs at the time of clinical visit when a primary care provider actually sees a patient in the office instead of a quarterly meeting for intervention”,
3. “It would be more practical if A-GPS report could be made self-explanatory (eg, data dictionary on the back page) and include current asthma medication”
4. “Trainees needed additional education and training for asthma guidelines and A-GPS”.

**References for Supplement**

1. National Asthma E, Prevention P. Expert Panel Report 3 (EPR-3): Guidelines for the Diagnosis and Management of Asthma-Summary Report 2007. J Allergy Clin Immunol 2007;120:S94-138.

2. Yunginger JW, Reed CE, O'Connell EJ, Melton LJ, 3rd, O'Fallon WM, Silverstein MD. A community-based study of the epidemiology of asthma. Incidence rates, 1964-1983. The American review of respiratory disease 1992;146:888-94.

3. Ducharme F, Dell S, Radhakrishnan D, et al. Diagnosis and management of asthma in preschoolers: A Canadian Thoracic Society and Canadian Paediatric Society position paper. Paediatrics & child health 2015;20:353 - 71.

4. Wi CI, Sohn S, Rolfes MC, et al. Application of a Natural Language Processing Algorithm to Asthma Ascertainment. An Automated Chart Review. American journal of respiratory and critical care medicine 2017;196:430-7.

5. Wi CI, Sohn S, Ali M, et al. Natural Language Processing for Asthma Ascertainment in Different Practice Settings. The journal of allergy and clinical immunology In practice 2018;6:126-31.

6. Castro-Rodriguez JA, Holberg CJ, Wright AL, Martinez FD. A clinical index to define risk of asthma in young children with recurrent wheezing. American journal of respiratory and critical care medicine 2000;162:1403-6.

7. Kaur H, Sohn S, Wi CI, et al. Automated chart review utilizing natural language processing algorithm for asthma predictive index. BMC Pulm Med 2018;18:34.

8. Sears MR, Johnston NW. Understanding the September asthma epidemic. J Allergy Clin Immunol 2007;120:526-9.

9. de Groot EP, Nijkamp A, Duiverman EJ, Brand PL. Allergic rhinitis is associated with poor asthma control in children with asthma. Thorax 2012;67:582-7.

10. Dixon AE, Holguin F, Sood A, et al. An official American Thoracic Society Workshop report: obesity and asthma. Proc Am Thorac Soc 2010;7:325-35.

11. Blackman JA, Gurka MJ. Developmental and behavioral comorbidities of asthma in children. J Dev Behav Pediatr 2007;28:92-9.

12. Harris MN, Lundien MC, Finnie DM, et al. Application of a novel socioeconomic measure using individual housing data in asthma research: an exploratory study. NPJ Prim Care Respir Med 2014;24:14018.

13. Juhn YJ, Beebe TJ, Finnie DM, et al. Development and initial testing of a new socioeconomic status measure based on housing data. J Urban Health 2011;88:933-44.

14. Laurent O, Pedrono G, Filleul L, et al. Influence of socioeconomic deprivation on the relation between air pollution and beta-agonist sales for asthma. Chest 2009;135:717-23.

15. Spira-Cohen A, Chen LC, Kendall M, Lall R, Thurston GD. Personal exposures to traffic-related air pollution and acute respiratory health among Bronx schoolchildren with asthma. Environ Health Perspect 2011;119:559-65.

16. Roy A, Sheffield P, Wong K, Trasande L. The effects of outdoor air pollutants on the costs of pediatric asthma hospitalizations in the United States, 1999 to 2007. Med Care 2011;49:810-7.

17. McCreanor J, Cullinan P, Nieuwenhuijsen MJ, et al. Respiratory effects of exposure to diesel traffic in persons with asthma. N Engl J Med 2007;357:2348-58.

18. Brauer M, Hoek G, Van Vliet P, et al. Air pollution from traffic and the development of respiratory infections and asthmatic and allergic symptoms in children. Am J Respir Crit Care Med 2002;166:1092-8.

19. Polosa R, Salvi S, Di Maria GU. Allergic susceptibility associated with diesel exhaust particle exposure: clear as mud. Arch Environ Health 2002;57:188-93.

20. Manchester-Neesvig JB, Schauer JJ, Cass GR. The distribution of particle-phase organic compounds in the atmosphere and their use for source apportionment during the Southern California Children's Health Study. J Air Waste Manag Assoc 2003;53:1065-79.

21. Reddel HK, Taylor DR, Bateman ED, et al. An official American Thoracic Society/European Respiratory Society statement: asthma control and exacerbations: standardizing endpoints for clinical asthma trials and clinical practice. Am J Respir Crit Care Med 2009;180:59-99.

22. Liu AH, Zeiger R, Sorkness C, et al. Development and cross-sectional validation of the Childhood Asthma Control Test. J Allergy Clin Immunol 2007;119:817-25.

23. Nathan RA, Sorkness CA, Kosinski M, et al. Development of the asthma control test: a survey for assessing asthma control. J Allergy Clin Immunol 2004;113:59-65.

24. Greenberg S. Asthma exacerbations: predisposing factors and prediction rules. Curr Opin Allergy Clin Immunol 2013;13:225-36.

25. Forno E, Celedon JC. Predicting asthma exacerbations in children. Curr Opin Pulm Med 2012;18:63-9.

26. Wolfson J, Bandyopadhyay S, Elidrisi M, et al. A Naive Bayes machine learning approach to risk prediction using censored, time-to-event data. Stat Med 2015;34:2941-57.

27. Assurance NCfQ. Technical Specifications for Health Plans. The Healthcare Effectiveness Data and Information Set (HEDIS)2016.

28. Visscher SL, Naessens JM, Yawn BP, Reinalda MS, Anderson SS, Borah BJ. Developing a standardized healthcare cost data warehouse. BMC Health Serv Res 2017;17:396.

**Verbal Consent Script**

**Topic of Telephone Script:**

This telephone script will be used to introduce the study and confirm the study eligibility before asking for participation in the study.

**Introduction:**

“Hello,” this is _________ calling from the Mayo Clinic in Rochester. May I please speak to _____________(or _____________’s parents or caregiver)?

****If the participant is available, continue with the script.*

***If the participant is not available, ask when it would be a good time to call to speak with ______________?

Describe the Reason for the Call:

We are contacting you today to ask you and your child to participate in our research project. Our research project is trying to provide better care to children with a history of breathing troubles or asthma. We are asking that participants complete a simple questionnaire, 5-7 questions, four times over a period of 12 months that looks at specific respiratory symptoms. Otherwise, there is nothing special you would need to do for this research. As a token of appreciation, upon completion of the study, we will send you a total of $20 or $5 for each completed report of the asthma questionnaire. Do you have any questions for us now? *** If the participant has questions, the staff addresses those. *** if the participant has not questions, the staff moves on.

**Describe in detail about study if parent asks.**

The goal of this project is to help your primary care provider deliver timely and improved care for children with breathing troubles or asthma , including better asthma management or the timely detection of asthma. As this project has to do with your primary care provider, you do not need to do anything except complete an asthma questionnaire every 3 months by your preferred choice (phone, paper form, or email). You may ask your child for their asthma symptoms and control status while filling out the questionnaire, if needed. To assess whether the study intervention makes a difference, we will assess respiratory health of your child every 3 months for one year by using age-appropriate asthma/respiratory symptom questionnaires, specifically the Asthma Control Test (ACT) and/or Test for Respiratory and Asthma Control in Kids (TRACK). To help you, we will send a reminder email or phone call quarterly with a link to the online version of one of these questionnaires which consists of 5-7 simple questions to complete.

Would you and your child be willing to participate and can I give you some more details about our research study?

(If Yes)

Briefly, after randomizing study participants, if your child happens to be in an intervention group, our research team provides relevant information obtained from your child’s medical records and other information sources to your primary care provider to better meet the needs of your child’s respiratory health (including the timely detection of asthma: Block 3 only). If your child is assigned to a control group, your child’s primary care provider will continue the current usual care. Therefore, this project will not affect any of your child’s usual care.

As you and your child do not need to do anything except completing the quarterly asthma questionnaire, there is no risk to you but a minimal risk of a potential breach of confidentiality. This will be prevented by adherence to our institutional policy.

Knowledge gained from this research will significantly improve respiratory care including asthma and outcomes for many children like yours through supporting your child’s primary care provider.

Please understand your participation is voluntary and you have the right to withdraw your consent or discontinue participation at any time without penalty. Specifically, your current or future medical care at the Mayo Clinic will not be jeopardized if you choose not to participate.

We will send you a letter and an authorization form to use protected health information (called HIPAA authorization form). This letter and HIPAA form will explain to you about the study purpose and design, and ask you to sign the form allowing us to use your child’s medical records for this study.

Closing

Thank you for participating in our research study. If you have any questions and changes, please contact us at either asthmaresearch@mayo.edu or 507-951-9296.

(If No)

Closing

(If the parent/patient is not interested in participating in the study, ask if they would like you to send the packet including letter explaining study in detail. If no, thank them for their time and discontinue the conversation).
