# Supplementary material for: Artificial intelligence-assisted clinical decision support for childhood asthma management: A randomized clinical trial
Source: PLoS One. 2021 Aug 2;16(8):e0255261. doi: 10.1371/journal.pone.0255261 (PMC8328289; doi:10.1371/journal.pone.0255261)
Supplement: S3 File — (PDF) [file pone.0255261.s003.pdf]

General Study Information

Principal Investigator: Young J. Juhn, M.D., MPH

Study Title: Enhancing Asthma Care and Outcome through the Implementation of asthma-Guidance and Prediction System (a-GPS) on Asthma Management Program: A Randomized Block Design

Protocol version number and date: Version 1, drafted at 06/19/2015

Purpose

A. SPECIFIC AIMS

Despite the availability of evidence-based guidelines for asthma management and effective asthma therapies, asthma continues to cause a significant morbidity and burden to our society. Growing deployments of Electronic Health Records (EHRs) systems have established **large practice-based longitudinal datasets,** which allow for the identification of patient cohorts for epidemiological investigations and population-based management. Natural Language Processing (NLP), which can extract structured information from narrative text (e.g., information on asthma symptoms and temporality during asthma evaluation by clinicians), has received great attention and has played a critical role in secondary use of EHRs for clinical care and translational research. For example, we recently developed an NLP algorithm for the Predetermined Asthma Criteria (PAC) that showed significantly improved sensitivity and specificity when compared to structured data such as ICD9 codes. At the same time, as addressed by President Obama at the State of Union address, **precision medicine,** which **identifies subgroups** of patients with differential susceptibility to diseases, biological processes, responses to therapies, and prognosis of disease based on patient-reported and objective data and **provide the optimal therapies guided and predicted by data**, is becoming a future model for health care.

In caring for childhood asthma, care coordination program is a widely implemented clinical approach to manage childhood asthma and typically includes patient education, care coordination, and enforcement of National Asthma Education and Practice Program (NAEPP) guidelines. While it has improved asthma outcomes and care quality, important concerns in the current asthma care by case management are 1) **the lack of tools enabling care coordination at a population level** despite the era of EHRs, **2) the lack of integrated data-driven surveillance system capturing temporal and geospatial trends of asthma outcomes and care,** and 3) **the resultant unguided resource allocation by data and inadequate optimization of asthma care.** As an example, we previously reported that two-thirds of children with asthma had a delay in their diagnosis (median years between asthma onset and diagnosis: 3.3 years), with subsequent asthma outcome and care largely ignored during the delayed period. Therefore, the current asthma care through care coordination strategy alone has significant constraints in delivering optimal care for children with asthma at a large scale including those without a diagnosis. This proposed study addresses these concerns and enhances asthma outcomes and care.

The primary goals of this proposed clinical trial are 1) to implement the asthma-Guidance and Prediction System (a-GPS) on Asthma Management Program (AMP, a current care coordination program for asthma care of children aged 5-17 years at Mayo Clinic) and 2) assess the impact of a-GPS on the primary and secondary end points for a one-year study period. **These goals** will be accomplished by conducting **a randomized clinical trial with block design** for three groups of children as the groups (blocks) of children are significantly heterogeneous in terms of receiving asthma care (Block 1. Persistent asthmatics aged 5-17 years enrolled in

AMP; Block 2. Persistent asthmatics who were not enrolled in AMP aged 0-17 years; and Block 3. Children with recurrent asthma-like symptoms within 3 years prior to the date of enrollment who do not have a documentation of a diagnosis of asthma in medical records aged 0-17 years), which compare the end points between the intervention group (usual care plus a-GPS) and control group (usual care only) in each block of children for the one-year study period.

The **a-GPS** program includes 1) **natural language processing** (NLP) capabilities (i.e., automated EHR review to identify asthma status (yes vs. no) and monitor asthma activity (onset, remission, and relapse) in real time), 2) **temporal and geospatial trends analysis** of asthma outcome and care, and 3) **asthma care optimization** through predictive analytics. **The primary end points** include asthma exacerbation defined by ER visit/hospitalization for asthma or unscheduled visit for asthma requiring oral corticosteroid, care quality (timely care in response to asthma-related events), and costs (total costs per member). For those in Block 3, the rate of a physician diagnosis of asthma during the study will be also compared between the intervention and control groups as a measure for quality care.

Specific Aim 1: To develop and implement a-GPS on the current asthma care by identifying children with persistent asthma through EHR search (n=1,900).
Hypothesis for Aim 1: The implementation of a-GPS in the current care is logistically feasible.
Specific Aim 2: To assess the primary end points between intervention and control group by conducting a RCT with a block design (n=300).
Hypothesis for Aim 2: Children enrolled in the intervention group have improved primary end points compared to a control group.

Expected Outcomes: The proposed study will: 1) **Address the current limitations of asthma care and enhance asthma outcomes and care** through AMP integrated with capabilities of NLP utilizing EHRs and real-time data surveillance system. 2) **Enable precision medicine for asthma care at a population level. 3) Enhance research capabilities for asthma** by improving efficiency for data collection at a large scale.

Subject Information – charts, records, images, or specimens are considered 'subjects'

Target accrual is the proposed number of subjects to be included in your study at your site. "Subjects" may include Mayo Clinic charts, records, or specimens, ***and/or*** *charts, records, or specimens received at Mayo Clinic from external sources for collaborating analysis by the investigator under this IRB application*:

Study subjects

Target accrual: Total number: 2,200 (1,900 for Aim 1 and 300 for Aim 2)

Our study subjects are children who receive medical care from Mayo Clinic pediatric practice. Mayo Clinic pediatric practice provides pediatric care to about two thirds of children in Olmsted County, Minnesota, (the remaining children are cared for by Olmsted Medical Center). Currently, about 2200 children with asthma are estimated to be cared for by Mayo Clinic pediatric practice (not including Family Medicine practice). Of these, about 385 children are estimated to be those with asthma <5 years of age and 1754 children are 5-17 years of age. Of the 1754 children with asthma, 376 children are currently enrolled in Asthma Management Program (AMP, care coordination program for asthma at Mayo Clinic). The current eligibility criteria for AMP include 1) ages 5-17 years, 2) persistent asthma, and 3) medical care in pediatric practice at Mayo Clinic.

1. Inclusion Criteria: **For children in Block 1 (n=100)**, they must be enrolled in AMP at the time of enrollment. **For children in Block 2 (n=100),** children aged 0-17 years old should have 1) a physician diagnosis of persistent asthma by NLP program for the list of physician diagnoses referring to persistent asthma, and/or 2) persistent asthma equivalent condition by either the Healthcare Effectiveness Data and Information Set (HEDIS; e.g., ER visit or hospitalization for asthma during the past 12 months) or the National Asthma Education and Prevention Program (NAEPP; e.g., ≥2 exacerbations requiring oral systemic corticosteroids in the past 6 months for children aged 0-4 years and 12 months for those aged ≥5 years), and/or who have physician diagnosis of asthma with controller medication (eg, inhaled corticosteroid) documented in the past 12 months, but they were not enrolled in AMP at the time of enrollment or during run-in period (n=100). **For children in Block 3 (n=100),** children must meet the criteria for asthma delineated in Table 1 for asthma and recurrent asthma-like symptoms, but do not have a documentation of a diagnosis of asthma in medical records aged 0-17 years. With the randomization protocol, the distribution of factors affecting asthma outcomes or quality of care should be similar between the control and intervention group. All subjects will be recruited during similar time period (eg, summer vacation), in a way that subjects are randomized into control and intervention group as subjects are enrolled. In this manner, both control and intervention group will be recruited at the *same time during the year* thus minimizing differences in seasonal influences on asthma outcome and care to differences in seasonality-related factors in the comparison groups.

2. Exclusion Criteria: 1) Non-Olmsted County residents, 2) children who are not enrolled in Mayo Clinic downtown pediatric practice, 3) No research authorization for using medical records for research, 4) Immunosuppressive therapy, 5) Conditions making asthma ascertainment difficult for Block 3 (pulmonary function tests that showed FEV_1 to be consistently below 50% predicted or diminished diffusion capacity, tracheobronchial foreign body at or about the incidence date of asthma, wheezing occurring only in response to anesthesia or medications, bullous emphysema or pulmonary fibrosis on chest radiograph, PiZZ $alpha_1$-antitrypsin, cystic fibrosis, other major chest disease such as severe kyphoscoliosis or bronchiectasis), and 6) children and their caregivers who decline to participate in the study.

☐ Yes ☒ No Will a Certificate of Confidentiality (COC) be obtained from NIH? If yes,
Who is obtaining the COC: Mayo Clinic investigator, study sponsor, other:
Explain why a COC is needed:

Study Design

Figure 6. Overview of the proposed Randomized Clinical Trial with a block design

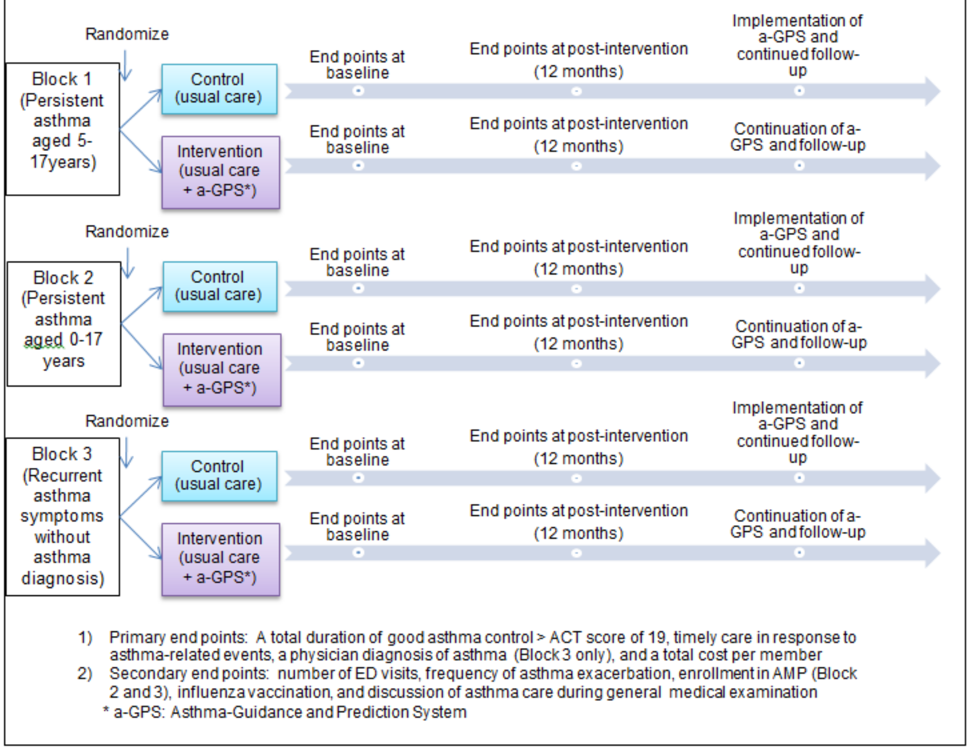

1. Study Population and Setting

Olmsted County, Minnesota, lies 90 miles southeast of Minneapolis, Minnesota. Rochester, Minnesota, is centrally located in Olmsted County. Characteristics of the City of Rochester and Olmsted County populations are similar to those of the U.S. Caucasian population, with the exception of a higher proportion of the working population are employed in the health care industry.[1-3] Contrary to perception and prior census results, the Rochester youth population is quite diverse with **22%** being classified as non-white in the 2010 census. The prevalence of childhood asthma (18%) is higher than national average.[4] Olmsted County, MN, is an excellent setting to conduct a population-based epidemiologic study because medical care is virtually self-contained within the community. Also, under the auspices of the Rochester Epidemiology Project (REP),[5] which has been continuously funded and maintained since 1960, each patient is assigned a unique identifier; all clinical diagnoses are electronically indexed, and information from every episode of care is contained within detailed patient-based medical records in all health care providers **(95%).**

2. Study Design: The overview of the study design is depicted in Figure 6 below.
Specific Aim 1: To develop and implement a-GPS on the current asthma care using retrospective chart review (n=1900).
Specific Aim 2: To assess the primary end points between intervention and control group by conducting a RCT with a block design (n=300).

2.1. Study intervention (a-GPS): 1) **automated electronic medical record review** program through natural language processing (NLP) to identify asthma status and monitor asthma activity (onset, remission, and relapse of asthma) in real time and structured data to detect poorly controlled asthma defined by Healthcare Effectiveness Data and Information Set (HEDIS) criteria; 2) temporal and geospatial **Trends analysis** for asthma-related data including the primary and secondary end points and other measures for asthma risk factors, outcome, and quality of care as summarized in Table 4 below; 3) asthma care optimization through feedback of processed information to care teams (clinicians, case managers, and community workers) by the study panel (asthma specialist, data analyst/retrieval specialist, NLP specialist, asthma care coordinator and project PI) to provide preventive and therapeutic interventions and recapture the short-term and long-term impact of such

interventions on risk reduction, outcomes, and care quality through steps 1 and 2. The information which will be retrieved and presented to clinicians for care optimization is listed in Table 5 with priority suggested by primary care pediatricians. Depending on priority, feasibility of data retrieval, and data quality, the final information to be provided to care team will be decided by the study advisory group.

Table 4. Variables to be collected (but not limited) for asthma risk factors, outcome, quality of care, and cost through a-GPS program

**Risk factors**	• Gender, race/ethnicity, family history of asthma, other atopic conditions (allergic rhinitis, eczema), household smoking exposure, pet at home, socioeconomic status (HOUSES, parental education)<br>• Probability of asthma exacerbation by NLP prediction model
**Asthma outcomes**	• Asthma Control Test (ACT) or Test for Respiratory and Asthma Control in Kids (TRACK) score<br>• The number of outpatient visits with asthma code (except well-child visits)<br>• ED visits or hospitalization with asthma code<br>• The frequency of exacerbation of asthma requiring systemic corticosteroids
**Quality of care**	• Enrollment in AMP (Block 2)<br>• Physician diagnosis of asthma (Block 3)<br>• Influenza vaccination<br>• Documented any asthma care after asthma-related events<br>• Appropriate medication for people with asthma (children with persistent asthma by HEDIS who were appropriately prescribed medication such as an inhaler)[6]<br>• Medication management for people with asthma (children with persistent asthma by HEDIS who remained on an asthma controller medication for at least 75% of their treatment period)[6]<br>• Optimal asthma care (children with persistent asthma who met all three targets, including: 1) well-controlled asthma based on ACT/ACQ/ATAQ score, AND 2) Total number of ER or hospitalization due to asthma less than 2 in last 12 months, AND 3) children who has been educated and has a written asthma management plan)[7]
**Cost**	• Average expenditures for asthma care per member per month<br>• A total cost of health care per member per month

2.2. Study Blocks: We will include three blocks for this RCT which are characterized by the nature and type of asthma and age group. The main rationale for the block (stratified) design is the significant heterogeneity of the three groups of children with asthma or asthma-like symptoms in terms of receiving asthma care affecting outcome measures.

1. Block 1. Children with persistent asthma aged 5-17 years enrolled in AMP (n=100)
2. Block 2. Children aged 0-17 years old should have 1) a diagnosis of persistent asthma by NLP program for the list of physician diagnoses referring to persistent asthma, and/or 2) persistent asthma equivalent condition by either the Healthcare Effectiveness Data and Information Set (HEDIS; e.g., ER visit or hospitalization for asthma during the past 12 months) or the National Asthma Education and Prevention Program (NAEPP; e.g., ≥2 exacerbations requiring oral systemic corticosteroids in the past 6 months for children aged 0-4 years and 12 months for those aged ≥5 years), and/or who have physician diagnosis of asthma with controller medication (eg, inhaled corticosteroid) documented in the past 12 months, but they were not enrolled in AMP at the time of enrollment or during run-in period (n=100)
3. Block 3. Children aged 0-17 years with recurrent asthma-like symptoms within 3 years prior to the date of enrollment who do not have a diagnosis of asthma (n=100)

2.3. Treatment groups: We will conduct **a randomized clinical trial with a block design** in two groups of children with persistent asthma or those with asthma-like symptoms, ages 0 and 17 years at enrollment, in Olmsted County, MN. In each block, we will randomize children into the following two treatment groups. Study subjects will receive the following treatments for a period of 12 months, either their:

1. Usual asthma care (a control group) (N = 150);
2. Usual asthma care plus a-GPS program (intervention group) (N = 150).

We will compare the primary and secondary end points at baseline (before intervention) and at 12 months post-intervention between the intervention and control group.

2.4. Primary End Points: The primary outcomes will be measured in the area of asthma outcome, quality of care, and costs during 12-month study period:

1. The number of ED visits/ hospitalization for asthma or unscheduled visit for asthma requiring oral corticosteroid (outcome): The number of ED visits/ hospitalization for asthma or unscheduled visit for asthma requiring oral corticosteroid will be retrieved from EHRs using NLP algorithm (from the Advanced Cohort Explorer system), and ICD (diagnosis for asthma exacerbation, 493.02, 493.12, 493.22, 493.82, and 493.92), CPT (office, ED visits, or

hospitalization), and national drug codes (for drugs). We already have algorithms to retrieve these data (see Preliminary Studies, C.1.3.3) and CPT codes for asthma and ED visits.

Table 5. Retrospective information/data to be retrieved and provided to care team for care optimization

Priority 1	Priority 2	Priority 3
Household smoking exposure<br>HOUSES<br>Hot spot for high traffic volume<br>Hospitalization for asthma in the past	ED visit for asthma<br>Family history of asthma<br>Other atopic conditions<br>Result of Spirometry<br>Unscheduled visit<br>Documentation of asthma control<br>Documentation of asthma severity<br>Influenza vaccine status<br>Systemic corticoid use<br>Appropriate medication for persistent asthma<br>Identification of persistent asthma<br>Result of allergy test<br>Result of AMP care	Documentation of asthma medication adherence<br>Enrollment of AMP<br>Missed school/work days<br>Pet at home<br>Change of asthma severity<br>Documentation of allergy/irritant assessment<br>Documented asthma care during GME<br>Documentation of inhaler technique<br>BMI<br>Comorbidity (infection)<br>Parental educational level<br>September epidemic<br>Documentation of asthma action plan used<br>Frequency of spirometry test<br>Frequency of scheduled asthma care visit

2. Timely care in response to asthma-related events (quality of care): Documented any asthma care after asthma-related events will be retrieved via NLP program and the Advanced Cohort Explorer system (a rich clinical data repository allowing in-real time search for terms in EHRs of millions of patients using text search functionality). NLP program will search term for Asthma Management Program as all asthma care through the current asthma care coordination documented under AMP in EHR). We will measure this end point as binary and the length of time between the events and asthma care delivered. Data will be retrieved from EHRs and verified by manual review for a subgroup of subjects.
3. A physician diagnosis of asthma (quality of care): This outcome will be limited to a group of children who had recurrent asthma symptoms within 3 years prior to the time of enrollment, but asthma diagnosis has not been documented (Block 3 only, see above). Predetermined Criteria for Asthma (PAC) as delineated in Table 1 in the Preliminary Studies section will be applied to ascertain asthma status by running NLP algorithm to determine whether one meets the criteria for asthma. A physician diagnosis of asthma will be assessed by ICD codes and NLP program searching a physician diagnosis of asthma.
4. A total cost per member: A total cost of health care per member during the study period will be calculated regardless of asthma status.

2.5. Secondary End Points: The secondary outcomes will be measured in the area of outcome and quality of care:

1. A total duration of good asthma control >ACT score of 19 (outcome): A quarterly asthma control status will be measured by administering Asthma Control Test (ACT) by an asthma care coordinator, care team or study coordinator over the phone, mail, or online ACT questionnaire with a reminding system using phone, email, text message (using our research cell phone (507-951-9296) or Internet website providing free text service), and Mayo portal if available. We will ask parents to fill out the questionnaire, but encourage them to get help from their child if needed.

2. Enrollment in AMP (quality of care): This outcome will be limited to a group of children (aged between 5 and 17 years) who had persistent asthma but were not enrolled in AMP (Block 2) or had recurrent asthma symptoms within 3 years prior to the date of enrollment but no physician diagnosis of asthma (Block 3). If they are enrolled in AMP during study period as a result of intervention (or usual care), they will not be censored but continue to be followed up until study ends, and analyzed according to the original stratified blocks (ie, intention to treat analysis)

3. Influenza vaccination (quality of care): Seasonal influenza vaccination status during the study period will be retrieved from EHRs using NLP from the Advanced Cohort Explorer system and CPT codes for influenza vaccination.
4. Discussion of asthma care during general medical examination (quality of care): We will retrieve documented asthma evaluation and care plan at general medical examination among subjects who have such visits during the study period from EHRs using NLP from the Advanced Cohort Explorer system.

3. Study procedures

3.1: Identification and random selection of study subjects: We have extensive retrospective and prospective research experiences with children with asthma in terms of identification and sample selection of children based on the inclusion and exclusion criteria as demonstrated in the Preliminary Studies section. We will have three blocks in terms of AMP enrollment status, asthma severity, and a physician diagnosis of asthma to ensure equal distribution of subjects to intervention and control groups with regard to these variables. **For Block 1,** we have already identified children ages 5-17 years who are currently enrolled in AMP from whom we will select a random sample of 100 children and adolescents based on the inclusion and exclusion criteria. Similarly, f**or Block 2,** we have already identified children with persistent asthma ages <18 years in pediatric practice at Mayo Clinic (~228) and will select an age and gender-stratified random sample of 100 children to ensure equal proportion of gender and each age representation. **For Block 3,** we have extensive experience with applying Predetermined Asthma Criteria to EHRs to identify children with asthma for asthma epidemiology research in our research work. We will select a random sample of 100 children who meet the Predetermined Asthma Criteria (Table 1) without a physician diagnosis of asthma.

3.2. Development of prediction model: We will develop a statistical machine learning model incorporating temporality to predict patient's asthma exacerbation based on a set of pre-defined risk factors, including socio-demographic factors, common clinical characteristics associated with asthma exacerbation recorded in structured data. Also, natural language processing techniques will extract risk factors resided in clinical narratives to augment the quality of the prediction model. This prediction model will also incorporate the information whether each subject belongs in hotspots for poor asthma control status. Prediction score for asthma exacerbation along with other asthma-related clinical information in individual's EHR will be given to primary care clinicians for asthma care intervention.

3.3. Recruitment and retention plan: The trial will be conducted in children, ages <18 years at the time of recruitment. Subjects will be recruited from the registry of children with asthma enrolled in the Mayo Clinic pediatric practice, who reside in Olmsted County, Minnesota, as described above. The Pediatric Asthma Epidemiology Research Unit (Dr. Juhn's research unit) and Asthma Management Program (care coordination program for asthma at Mayo Clinic) have an extensive experience with recruitment of children from the community for asthma research and care in the past. As noted in the preliminary studies section, as of now, we have identified about 2200 children with asthma ages < 18 years who are potentially available and eligible for this trial. The recruitment plan, with attention to accent and consent issues, is formally discussed in the PROTECTION OF HUMAN SUBJECTS section. Recruitment will be carried out by Dr. Juhn, Ms. Joy Green (leader of AMP) and the designated nurse coordinators.

3.4. Allocation of intervention (randomization) and study intervention: Fifty interventions and fifty controls will be ranked in a random order and this rank will be assigned to the eligible hundred participants per block. Gender and age (within 1 year) will be the only stratification factor to ensure that the treatment groups are balanced within gender and age categories (0-3, 4-6, 7-10, 11-13, and 13-17 years of age). As this study is not blinded, both parents and children will be informed about the nature of intervention and control group care and this study (intervention) will not affect the current workflow of usual care for asthma including AMP. Once subjects are

allocated to control group, children in control group will receive usual care for asthma including AMP. For subjects allocated to intervention group, the intervention will take place by supporting the study coordinator receiving processed and interpreted data for a-GPS from the study panel about children allocated to intervention group on a quarterly basis. A designated study coordinator, care team or study coordinator will have a designated time for asthma care through a-GPS and usual care. As happens in usual asthma care, all preventive and therapeutic treatments for asthma will be made by a care team including a designated nurse coordinator, patients' primary care providers, and community health worker. The intervention group will have a higher quality data for asthma in real time which will be available to the care team for any changes of asthma care. For example, for Block 3, when a group of children, who meet PAC, are identified, a nurse coordinator will inform primary care clinician for a patient about this information (ie, evidence for asthma, information with the narrative statements in EHRs with the documented dates showing why the patient meets the PAC) and a primary care clinician will make a decision whether the clinician will diagnose the patient with asthma. Similarly, for Block 1 and 2, similar outcomes or quality of care data are provided to a care coordinator, care team or study coordinator for changes of asthma management based on the provided data.

3.5. Measurement of the end points: The end points described above will be measured after one-year study period. Outcome measure will be performed using various data pulling mechanism including NLP and structured data from various data sources, which are being supplied by EHRs.

3.6. Safety Monitoring: Although this proposed study is a RCT, it does not interfere the usual care for asthma. The intervention is provision of asthma-related information (outcome, quality of care, and risk factors) to a designated study care coordinator for asthma. Any changes of clinical care for asthma for patients will be made through clinical care team. Therefore, participating in the study is not posing a risk to patients except breach of confidentiality. Details about risk and protecting subjects for potential risk are discussed in the Human Subject section.

4. Data Analysis

Baseline demographic and clinical features will be compared between children in the intervention and control groups using two-sample t, Wilcoxon, chi-square, and Fisher exact tests to identify features that are not balanced between the two groups at baseline. Detailed analytic approaches comparing post-intervention outcomes will be presented below, following approaches described in preliminary studies. The full details of the analysis plan will be fully enumerated in the statistical section of the protocol developed for IRB submission and PI and study statistician will oversee it.

For the total duration of good asthma control >ACT score of 19 for children ≥ 4 years or <TRACK score of 80 for children <4 years on a quarterly basis, which is one of the secondary end points, we will measure this outcome as a number of recurrent events per 12-month periods (proportion of events per person-months). We will fit the ANOVA F-test by treating blocks as fixed effects to examine the association between two groups (intervention vs control) and this outcome. For the timely care in response to asthma-related events, we will measure this outcome in a binary (timely care occur yes vs. no) and a continuous variable (the duration between asthma-related events and care intervention). For the binary variable, we will perform conditional logistic regression model to estimate the parameters (odds ratios and 95% CI) for the association between intervention and this outcome and for the continuous variable, we will perform the ANOVA F-test with fixed block effects. For total costs per member, it will be measured in a continuous variable and the analysis will be similar to continuous variables described above. For a physician diagnosis of asthma (limited to Block 3 only), similarly, we will measure this primary end point in a binary (delayed diagnosis occur yes vs. no) and a continuous variable (the duration between index date when one meets the PAC and the date of physician diagnosis). Logistic regression for binary variable and linear regression for continuous variable will be performed to test the difference between two groups.

The analytic approaches for other end points including asthma exacerbation defined by the number of ED visits/hospitalization (incidence density, number of events per 12 months) or the frequency of exacerbation of

asthma requiring systemic corticosteroids (incidence density, number of events per 12 months), the frequency of the referral to AMP (limited to Block 2 and 3 only), influenza vaccination (binary outcome), and discussion of asthma care during general medical evaluation (binary outcome) will be similar to those for other outcomes described above.

The fundamental analysis principle will be **Intention to Treat,** accounting for all participants randomized in the primary analysis. It is likely we will have missing outcome data over the 12-month treatment period, despite efforts to minimize this. This missing is considered missing completely at random, so the effect of the missingness patterns is expected to be minor in this study. Also, there might be some patients in Block 2 or 3 who could be referred to AMP during the study period as a result of intervention and/or usual care, but we will not censor those subjects but follow them through study period with considered as original Block (i.e., 2 or 3).

5. Sample Size and Power Estimation

The sample size is estimated using the two-way ANOVA (equal variance). As we do not have the data on the total duration of good asthma control > ACT score of 19 or TRACK scores < 80, the power calculation is based on the reported data from the literature. A recent clinical trial showed that children with persistent asthma had 9.5 symptom free days per 2 week period (about 70% of time and estimated standard deviation of 2.6 days, i.e., 8.4 months per year).[8] If we assume that the durations of good asthma control for children in Block 1, those in Block 2, and those in Block 3 who receive usual care (control group) are 9, 8.4, and 10 months, given the sample size (50 children per Block in each treatment group), we will have 80% power to detect the corresponding differences **(7-11%)** in intervention group, 10, 9, and 11 months, respectively. For the timely care in response to asthma-related events, similarly, if we assume the durations between asthma events and asthma care intervention are 7 days for Block 1, 30 days for Block 2, and 90 days for Block 3 in usual care group (control group) and common standard deviation of 8.2 days, we will have 80% power to detect **the differences (~5%)** in intervention group, 6, 28, and 85 days, respectively. For a physician diagnosis of asthma for children who meet PAC, our pilot data showed 64% of children who met PAC did not have a physician diagnosis in the current asthma care, if we assume this is true for control group in our study, given the sample size (50 per intervention and 50 per control group), we will have 81% power to detect odds ratio of **0.29** (reducing the odds of not being diagnosed in intervention group). For the total health care cost per member per month, a recent study reported the total health care costs per member per 100 children in usual care for children with persistent asthma was $13,574 (i.e., $136 per member per month).[9] If we assume that the total health care costs per member per month for children in Block 1, Block2, and Block 3 are $136, $150, and $120, respectively in control group and common standard deviation of $21.5, we will have 82% power to detect **about 5%** differences in the reduced costs in intervention group, $129, $142, and $114, respectively. Therefore, this trial has adequate power to address the study aims.

6. Data Monitoring Plan

Patient safety will be monitored per the safety protocol for clinical trials at Mayo Clinic. We do not anticipate any specific adverse events during the study as the intervention is informational, not procedures or medication supplement. Therefore, we do not believe the Data Monitoring Safety Board (DSMB) needs to be in place for this proposed study but will obtain the guidance from the IRB. There will be periodic interim analysis to evaluate study progress, safety data quality if necessary, and protocol compliance and baseline covariates will be included, comparing the treatment groups as well as describing the participants enrolled. There will be interim analyses for the primary and secondary outcomes at 6 months for quality control purpose. These results will not be used for premature termination of the study since the outcomes are not clinical events. However, group sequential methods for monitoring safety are proposed, subject to confirmation by the DSMB. We are proposing a Pocock [10] type □ spending function (0.025 significance level, one sided) for the lower boundary to monitor for harm at each of the interim analyses. This lower boundary is not binding but rather a guideline for the DSMB, especially when evaluating Serious Adverse Events (SAEs). The DSMB will be guided by a charter similar to the one published by Ellenberg, Fleming and DeMets [11].

Check all that apply. If none apply, leave blank:

☐ This is a multisite study involving Mayo Clinic and non-Mayo Clinic sites.
When checked, describe the research procedures/activities being conducted **only** at Mayo Clinic:

☐ Mayo Clinic staff will be engaged in research activity at a non-Mayo Clinic site. *When checked, provide the location and a detailed description of the Mayo Clinic research staff involvement.*

☒ This study is to establish and/or maintain an ongoing database or registry for research purposes only.

☒ The research involves contact or interaction with subjects, for example, surveys, questionnaires, observation, blood draw.

☐ The study involves photographing, audiotaping or videotaping subjects (and guests).

PROTECTION OF HUMAN SUBJECTS

Proposed Involvement of Human Subjects.

All subjects will be children ages <18 years and they will be identified and recruited from the registries of AMP and Mayo Clinic pediatric practice. Recruitment procedures are described below.

Sources of Research Material

Research material will be obtained from individually identifiable living human subjects through interview and EHRs as described in Research Design and Methods. All such data collected will be obtained specifically for research purposes.

Safety Monitoring: Please see the Safety Monitoring section in Study Design.

Recruitment of Subjects and Consent-and-Screening Procedures

Children will be recruited from the registry of Mayo Clinic pediatric practice. The research study coordinator at Mayo Clinic Rochester will review medical records to screen eligible candidates. Then, we will contact the eligible children and their parents/guardians by telephone (with a maximum of three) or a recruitment letter explaining study objectives and procedures (ie, providing AMP coordinator with results of medical chart review before and during study period) and obtain verbal consent. There is no specific requirement for parents or patients for this specific study apart from usual clinical care for asthma. We will send a letter to the parents/guardian of the eligible subjects who wish to participate in the study as a part of verbal consent. This letter will contain a postcard addressed to the study coordinator that is to be mailed back with their signature for HIPPA authorization within two weeks. We are sending a reminder to those who did not resend us HIPAA form after verbal consent (eg, email, phone, text message). Additionally, we may send a recruitment (introduction) letter with HIPAA form to the parents/guardians before calling parents/guardian, to make timely recruitment, if needed. After receiving the signed postcard and HIPPA form, the study coordinator will confirm the inclusion criterion. The exclusion criteria are listed in the main protocol.

Potential risks

The only risk involved with this study is breach of confidentiality. Risks for breach of confidentiality through review of medical records will be prevented by adherence to our institutional policy.

Procedures for Protecting Against or Minimizing Potential Risks

Data safety: Verbal consent (signed HIPPA form) for study participation and study data apart from clinical data or EHRs will be stored in secured files, either in locked file cabinets or in a locked room. Only investigators or individuals authorized by the project staff will have access to a database. Study data will be managed by assigned subject identification numbers or medical record numbers and analyzed anonymously. The identifiers will be destroyed at the conclusion of the research work. Subsequent reports will be of a summary nature with no identifiable patient data. No medical records will be reviewed from any patients for whom the general research authorization has been refused.

Confidentiality of all subjects participating in the proposed research will be fully protected. All study records apart from clinical data or EHRs are kept in locked file cabinets. Only a unique study number, which bears no relationship to personal identifiers including name, initials, address, telephone number, social security number, or patient number identifies individual subjects in all computer files and all analyses. Consequently, depositing such "anonymous" study data and results into the data sets should encounter no concerns regarding the protection of human subjects. Email correspondences for receiving their ACT or TRACK score back will be deleted after transferring the scores to data abstraction form which will be kept in locked file cabinets.

Risk-Benefit Balance
The potential risks described above are reasonable in relation to the importance of the knowledge that may be gained. Knowledge gained from the proposed research has the potential to improve asthma care and outcome through innovative informatics approaches.

Inclusion of Children
All study subjects will be children ages <18 years at the time of enrollment who receive medical care at Mayo Clinic pediatric practice because informatics program (ACE) is only available at Mayo Clinic in the community. Thus, we do not exclude children from this study.

Inclusion of Women and Minorities
We will enroll all eligible children who are currently enrolled in the Mayo Clinic pediatric practice. Thus, this study does not exclude female subjects. Minorities will be included in this study. Currently, 22% of children in Olmsted County, MN are minority children and we anticipate our study sample is likely to reflect this ethnic proportion of minorities among children.

References

1. Census Bureau, 1980 and 1990 Census of Population and Housing. Washington D.C.1983 and 1993.
2. Katusic SK, Colligan RC, Barbaresi WJ, Schaid DJ, Jacobsen SJ. Potential influence of migration bias in birth cohort studies. Mayo Clinic Proceedings 1998;73:1053-61.
3. Yunginger JW, Reed CE, O'Connell EJ, Melton LJ, 3rd, O'Fallon WM, Silverstein MD. A community-based study of the epidemiology of asthma. Incidence rates, 1964-1983. Am Rev Respir Dis 1992;146:888-94.
4. Yawn BP, Wollan P, Kurland M, Scanlon P. A longitudinal study of the prevalence of asthma in a community population of school-age children. Journal of Pediatrics 2002;140:576-81.
5. Kurland LT, Molgaard CA. The patient record in epidemiology. Sci Am 1981;245:54-63.
6. The State of Health Care Quality 2014, National Committee for Quality Assurance (NCQA), The Healthcare Effectiveness Data and Information Set (HEDIS) (Accessed November 27, 2014, at http://www.ncqa.org/ReportCards/HealthPlans/StateofHealthCareQuality.aspx.)
7. 2013 Minnesota Health Care Quarlity Report, Minnesota Department of Health. (Accessed December 23, 2014, at http://mncm.org/wp-content/uploads/2014/02/2013-HCQR-Final-2.4.2014.pdf.)
8. Krieger J, Takaro TK, Song L, Beaudet N, Edwards K. A Randomized Controlled Trial of Asthma Self-management Support Comparing Clinic-Based Nurses and In-Home Community Health Workers: The Seattle-King County Healthy Homes II Project. Arch Pediatr Adolesc Med 2009;163:141-9.
9. Noyes K, Bajorska A, Fisher S, Sauer J, Fagnano M, Halterman JS. Cost-Effectiveness of the School-Based Asthma Therapy (SBAT) Program. Pediatrics 2013;131:e709-e17.
10. Lan KKG, DeMets DL. Discrete sequential boundaries for clinical trials. Biometrika 1983;70;3:659-63.
11. Ellenberg S, Fleming T, DeMets D. Data Monitoring Committees in Clinical Trials, A Practical Perspective: Wiley.; 2002.
